# Blocking STAT3 signaling augments MEK/ERK inhibitor efficacy in esophageal squamous cell carcinoma

**DOI:** 10.1038/s41419-022-04941-3

**Published:** 2022-05-25

**Authors:** Zhen-Yuan Zheng, Man-Yu Chu, Wan Lin, Ya-Qi Zheng, Xiu-E Xu, Yang Chen, Lian-Di Liao, Zhi-Yong Wu, Shao-Hong Wang, En-Min Li, Li-Yan Xu

**Affiliations:** 1 The Key Laboratory of Molecular Biology for High Cancer Incidence Coastal Chaoshan Area, Department of Biochemistry and Molecular Biology, Shantou University Medical College, Shantou, 515041 Guangdong China; 2 Guangdong Provincial Key Laboratory of Infectious Diseases and Molecular Immunopathology, Institute of Oncologic Pathology, Shantou University Medical College, Shantou University Medical College, Shantou, 515041 Guangdong China; 3 Guangdong Esophageal Cancer Research Institute, Shantou Sub-center, Cancer Research Center, Shantou University Medical College, Shantou, 515041 Guangdong China; 4 Shantou Central Hospital, Affiliated Shantou Hospital of Sun Yat-Sen University, Shantou, 515041 Guangdong China

**Keywords:** Targeted therapies, Senescence

## Abstract

Esophageal squamous cell carcinoma (ESCC) is one of the world’s leading causes of death, and its primary clinical therapy relies on surgical resection, chemotherapy, radiotherapy, and chemoradiotherapy. Although the genomic features and clinical significance of ESCC have been identified, the outcomes of targeted therapies are still unsatisfactory. Here, we demonstrate that mitogen-activated protein kinase (MAPK) signaling is highly activated and associated with poor prognosis in patients with ESCC. Mitogen-activated protein kinase kinase (MEK) inhibitors efficiently blocked the phosphorylation of extracellular signal-regulated kinase 1/2 (ERK1/2) in ESCC, while signal transducer and activator of transcription 3 (STAT3) signaling was rapidly activated. Combined STAT3 inhibition prevented the emergence of resistance and enhanced MEK inhibitor-induced cell cycle arrest and senescence in vitro and in vivo. Mechanistic studies revealed that the suppressor of cytokine signaling 3 (SOCS3) was downregulated, resulting in an increase in STAT3 phosphorylation in MEK-inhibited cells. Furthermore, chromatin immunoprecipitation showed that ELK1, which was activated by MEK/ERK signaling, induced SOCS3 transcription. These data suggest that the development of combined MEK and STAT3 inhibition could be a useful strategy in ESCC targeted therapy.

## Introduction

Esophageal cancer (EC) is the sixth leading cause of cancer-related mortality, with a 5-year survival rate of <25% [1, 2]. Histologically, esophageal squamous cell carcinoma (ESCC) is the most common form of EC, accounting for almost 88% of cases worldwide [3]. Surgery, chemotherapy, radiotherapy, and chemoradiotherapy are the mainstays of ESCC treatment, but recurrence and cytotoxicity remain significant challenges [4]. Targeted therapy is more effective and has fewer adverse reactions [5, 6]. However, despite a great number of genomic studies suggesting potential biomarkers for targeted therapy, only a few biomarkers have been successfully used for targeted therapy [7, 8]. Therefore, there is an urgent need to integrate current data to optimize the potential therapeutic targets for ESCC.

Several groups have mapped genomic alterations in ESCC, and found that the receptor tyrosine kinase (RTK)-mitogen-activated protein kinase (MAPK) pathway is frequently dysregulated; 18.3 and 11.8% of patients with ESCC show overexpression of epidermal growth factor receptor (EGFR) and fibroblast growth factor receptors (FGFR) [8–11]. Mitogen-activated protein kinase kinase (MEK)/extracellular signal-regulated kinase (ERK) signaling is downstream of multiple RTKs and is highly activated in ESCC [12–14]. It has also been shown that phosphorylated ERK (pERK) is highly expressed and may be a possible marker for personalized treatment of EC [12]. Recently, blocking the MEK/ERK pathway via small molecule inhibitors was shown to effectively inhibit the growth of KRAS-mutant lung cancer and melanoma [15, 16]. Oncogene-activating mutations, including mutations in EGFR, KRAS, and BRAF, also confer susceptibility to MEK inhibitors in ESCC [12]. This suggests that MEK/ERK signaling may be a potential target for ESCC therapy. Although research suggests a target role for ERK signaling in ESCC therapy, there is no clinical trial to support the use of MEK inhibitors in the clinic [17, 18].

In diverse cancer models, MEK inhibition (MEKi) has shown limited to no effect due to the activation of compensatory pathways [19, 20]. For example, multiple RTKs and/or their ligands acquire resistance to MEKi in KRAS-mutant cancers [15, 21, 22]. This shows that MEKi may play a role in therapy, especially in combination therapy. MEKi can enhance the efficacy of EGFR inhibitors following adaptive resistance to EGFR blockade in ESCC [18]. Inhibition of the protein tyrosine phosphatase SHP2, a positive signal transducer between RTKs and RAS, can enhance the sensitivity of KRAS-amplified gastroesophageal cancer cells to MEKi [23]. These studies demonstrate that more MEKi resistance mechanisms and compensatory pathways need to be identified in ESCC.

Cellular senescence is a stress response that leads to cell cycle arrest, metabolic reprogramming, and senescence-associated secretory phenotype (SASP). Senescence can be induced by different stimuli, such as oncogene signaling, DNA damage, and oxidative stress [24]. It has been reported that MEK therapy combined with cyclin-dependent kinase 4/6 (CDK4/6) inhibition promotes a proangiogenic SASP in KRAS-mutant pancreatic ductal adenocarcinoma [25]. SASP components induced by MEK and CDK4/6 combination inhibition synergistically inhibit tumor progression through natural killer cell-mediated cytotoxicity in C57BL/6 immunocompetent mice injected with *Kras*^G12D/+^; *Trp53*^–/–^(KP) lung tumors [26]. Inhibition of ERK and AKT signaling also induces cell cycle arrest and senescence in several cancers [27–29]. Therefore, combination drug therapies that induce senescence have shown important benefits as anti-cancer agents.

Here, we sought to identify targets to augment the efficacy of MEKi in ESCC. Through reporter gene screening, we identified signaling transduction and activation transcription 3 (STAT3) activation as a novel compensatory pathway for the potential therapeutic resistance of ESCC to ERK signaling inhibition. We demonstrated marked efficacy of combined MEK and STAT3 inhibition (STAT3i) in vitro and in vivo. These findings suggest that the treatment efficiency of ESCC can be improved by blocking the two signaling pathways.

## Results

### Inhibition of ERK signaling activates the STAT3 pathway in ESCC

**Fig. 1:**
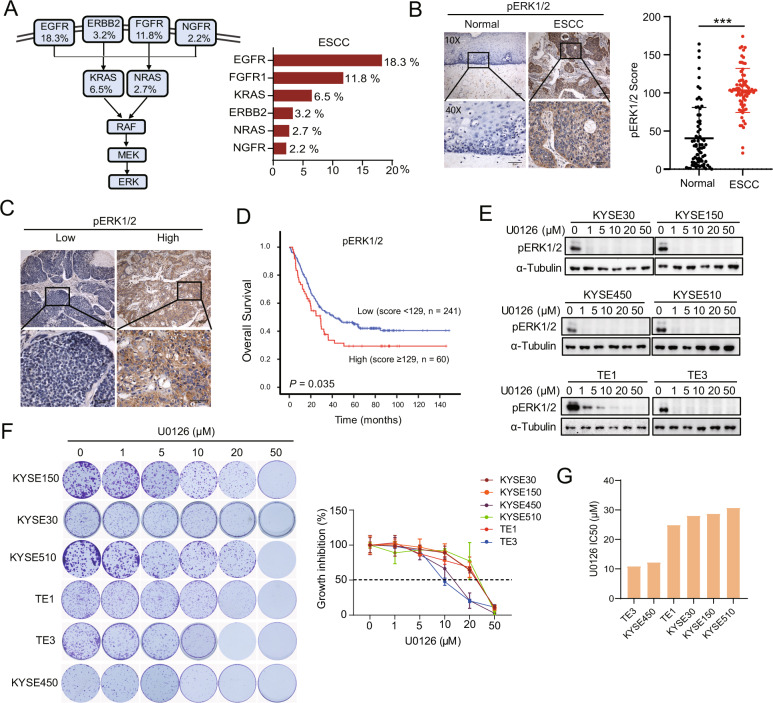
Hyperactive phospho-ERK (pERK) in patients with ESCC is associated with poor prognosis. **A** Frequencies of upstream mitogen-activated protein kinase kinase (MEK)/extracellular signal-regulated kinase (ERK) signaling-related gene amplification (*EGFR, ERBB2, FGFR, NGFR, KRAS*, and *NRAS*) in ESCC from The Cancer Genome Atlas. **B** pERK immunohistochemistry (IHC) in tumor and para-carcinoma tissues of patients with ESCC. Representative images of pERK expression in normal and ESCC tissues (*n* = 72) and statistical results. Scale bar, 100 μm. Data are presented as the mean ± standard deviation (SD). ***P < 0.001. **C** Representative low and high expression of pERK in tumor tissues obtained using IHC. **D** Kaplan–Meier survival curve of overall survival based on the expression of pERK (*n* = 301) in patients with ESCC. **E** Western blotting shows pERK expression after different concentrations of U0126 treatment in six ESCC cell lines. **F** Colony formation assays for six ESCC cell lines after different concentrations of U0126 treatment for 7–10 days. The growth inhibition ratio was calculated according to the number of colonies formed. **G** U0126 drug sensitivity IC50 values in six ESCC cell lines.

We examined the mutation status of RTK- and RAS-encoding genes, which might be attributed to ERK pathway activation as observed in TCGA. We observed alterations in MAPK pathway-related gene amplification (specifically, *EGFR, FGFR1, KRAS, ERBB2, NRAS*, and *NGFR*) in patients with ESCC (Fig. 1A). Immunohistochemistry (IHC) of pERK1/2 was performed with 72 pairs of tumor and para-carcinoma tissues from patients with ESCC. pERK1/2 staining was barely detectable in normal para-carcinoma tissues (mean score 40.4), while tumor tissues (mean score 103.2) exhibited a more diffuse pERK1/2 staining (Fig. 1B). Consistently, the high pERK1/2 expression level was associated with poor prognosis for overall survival in 301 patients with ESCC (*P* = 0.035) (Fig. 1C, D). We then evaluated the effect of ERK1/2 inhibition in six ESCC cell lines using U0126 (a MEK1/2 inhibitor). Phosphorylation of ERK1/2 was dramatically reduced or disappeared after treatment with 1 μM U0126 in all cell lines (Fig. 1E); however, as shown in Fig. 1F, ESCC cell lines exhibited resistance to 1 μM U0126, with a half-maximal inhibitory concentration (IC50) ranging from 10 to 30 μM (Fig. 1G). These results demonstrate that blocking ERK signaling is insufficient to inhibit ESCC cell growth.

**Fig. 2:**
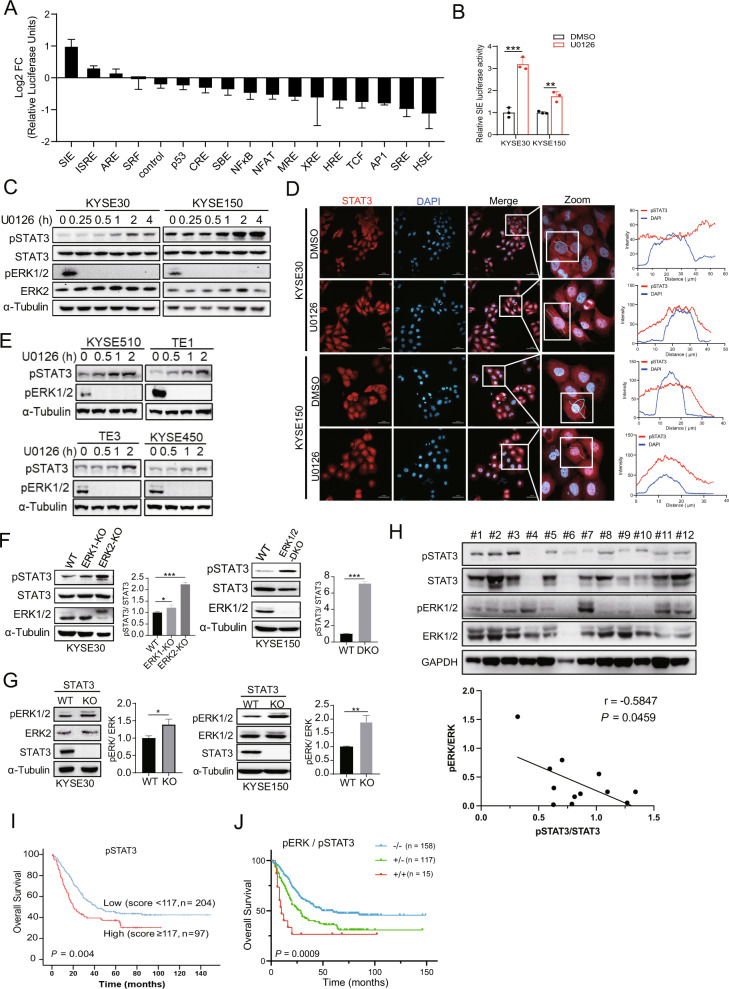
Inverse correlation of pERK and pSTAT3 expression in ESCC. **A** KYSE150 cells were transfected with luciferase reporter genes of 16 tumor-associated signaling pathways and renilla luciferase for 24 h. Cells were incubated with DMSO or U0126 (5 μM) for 6 h and luciferase activity was measured using Dual-Luciferase Assay. Log2 FC was assessed by dividing the luciferase signal obtained from U0126 treated cells by that obtained from DMSO-treated ones. Error bars represent the mean ± SD. **B** KYSE30 and KYSE150 were transfected with SIE and renilla luciferase reporter for 24 h. Cells were incubated with DMSO or U0126 (5 μM) for 6 h and luciferase activity was measured. **C** Western blotting showed the expression of activated and total signal transducer and activator of transcription 3 (STAT3) and ERK expression in KYSE30 and KYSE150 treated with U0126 in a time-dependent manner. **D** STAT3 and DAPI staining in KYSE30 and KYSE150 after U0126 (5 μM) treatment for 2 h. The fluorescence signal intensity of STAT3 and DAPI was analyzed using the ZEN2.3 software. Scale bar, 50 μm. **E** Western blotting showed the expression of pSTAT3 and pERK in KYSE450, KYSE510, TE1 and TE3 treated with U0126 in a time-dependent manner. **F** Western blotting shows the expression of pSTAT3 in wild-type (WT), ERK1-KO, ERK2-KO, and ERK1/2-DKO ESCC cells. **G** Western blotting shows the expression of pERK in WT and STAT3-knockout (KO) cells. Densitometry analyses of pSTAT3 or pERK expression were performed. Error bars represent the mean ± SD. *P < 0.05, **P < 0.01, ***P < 0.001. **H** Western blotting showed the phosphorylated and total STAT3 and ERK expression in 12 human ESCC tissue. Densitometry analyses and Pearson correlation showed a negative correlation for pSTAT3 and pERK expression. **I** Kaplan–Meier analysis was performed to evaluate the effects of pSTAT3 expression on overall survival (*n* = 301). **J** Kaplan–Meier analysis was performed to evaluate the effects of pERK associated with pSTAT3 expression on overall survival (*n* = 290). Group +/+: dual high expression level of pERK and pSTAT3; group +/−: high expression of pERK and low expression of pSTAT3 or low expression of pERK and high expression of pSTAT3; group −/−: dual low expression of pERK and pSTAT3.

Next, we investigated whether there is a compensatory mechanism induced by U0126. We examined gene alterations using 16 cancer-related signaling pathway reporters (Supplementary Table S1). U0126 treatment upregulated the luciferase activities of the SIE and ISRE reporters, which are STAT3 and STAT1/STAT2 binding motifs, respectively (Fig. 2A). The SIE luciferase activity increased after U0126 treatment in KYSE30 and KYSE150 cells (Fig. 2B). Therefore, we determined whether U0126 could activate STAT3 in ESCC cell lines. As expected, U0126 increased STAT3^Y705^ phosphorylation and nuclear localization after 2 h of treatment (Fig. 2C, D). Similar results were found in KYSE450, KYSE510, TE1, and TE3 ESCC cell lines (Fig. 2E). CRISPR-Cas9 knockout (KO) of ERK2 or ERK1/2-double knockout (DKO) resulted in increased levels of pSTAT3 (Fig. 2F). In addition, STAT3-KO cells showed increased levels of pERK1/2 (Fig. 2G). Next, we investigated the phosphorylation levels of STAT3 and ERK1/2 in 12 ESCC tissue and the results confirmed that the expression of pERK1/2 and pSTAT3 had an inverse correlation (*r* = −0.5847, *P* = 0.0459) (Fig. 2H). To further evaluate the prognostic value of pSTAT3 in a clinical setting, we performed a survival analysis of patients with ESCC. High expression of pSTAT3 resulted in a shorter median overall survival than low expression (*P* = 0.004, Fig. 2I). Using a risk score calculation, we combined pERK1/2 and pSTAT3 to predict the clinical outcomes of patients with ESCC. High expression levels of both pERK1/2 and pSTAT3 (group +/+) were strongly associated with shorter overall survival (*P* = 0.0009, Fig. 2J). These results supported that both inhibiting ERK signaling pathway and blocking STAT3 activity could prolong ESCC patient survival.

### Dual inhibition of MEK and STAT3 signaling decreases the proliferation of ESCC cells

**Fig. 3:**
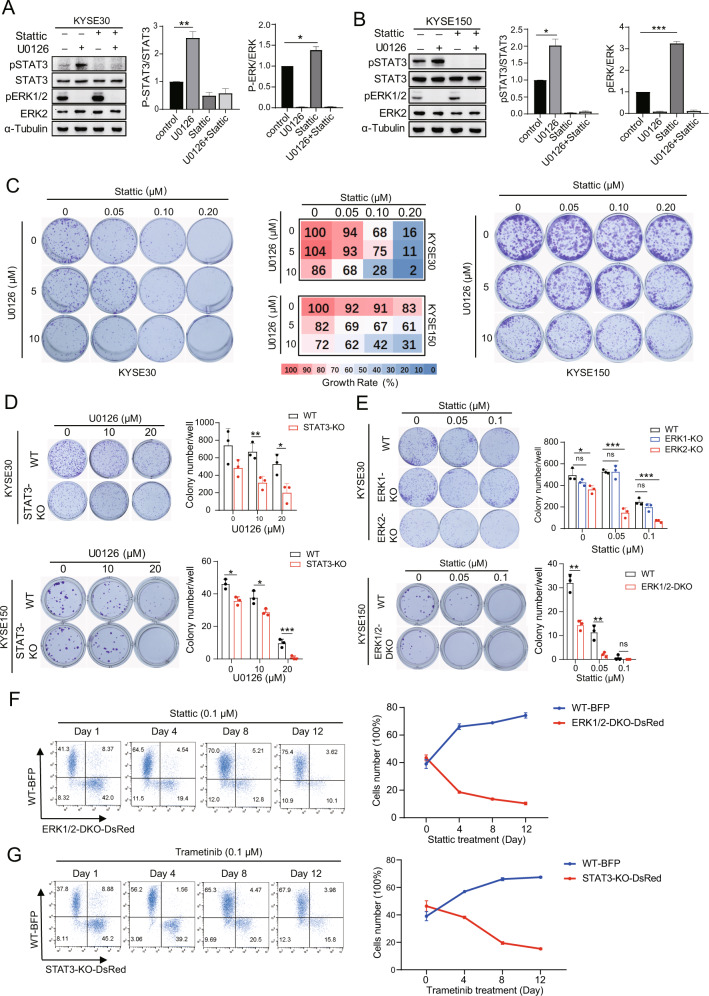
Dual inhibition of ERK and STAT3 signaling decreases the proliferation of ESCC cells. **A**, **B** Western blotting shows the expression of pSTAT3 and pERK in KYSE30 (**A**) and KYSE150 (**B**) after treatment with U0126 (5 μM) and/or Stattic (1 μM) for 4 h. Densitometry analyses of pSTAT3 or pERK expression are shown. Error bars represent the mean ± SD. *P < 0.05, **P < 0.01, ***P < 0.001. **C** Colony formation assays of KYSE30 and KYSE150 cells treated with a combination of U0126 and Stattic at different concentrations as indicated. The percentage growth at each inhibitor concentration is presented (middle). Data are presented as the mean of three independent experiments. **D**, **E** Colony formation assays of KYSE30 and KYSE150 STAT3-KO (**D**) and ERK1/2-KO (**E**) cells treated with U0126 at different concentrations, showing the colony formation number (means of three independent experiments). Error bars represent the mean ± SD. *P < 0.05, **P < 0.01, ***P < 0.001. **F** KYSE30 WT cells were stably transduced with EV-BFP (WT-BFP), and KYSE30 ERK1/2-DKO cells with EV-DsRed (DKO1/2-DsRed). Cells were mixed at equal numbers and treated with Stattic (0.1 μM). **G** KYSE30 WT cells were stably transduced with EV-BFP (WT-BFP), and KYSE30 STAT3-KO with EV-DsRed (STAT3-KO-DsRed). Cells were mixed at equal numbers and treated with trametinib (0.1 μM). Flow cytometry was performed to monitor the proportions of BFP + and DsRed+ cells at various time points. Shown are representative FACS plots from three independent experiments. Error bars represent the mean ± SD.

To evaluate the compensatory effect of pERK1/2 and pSTAT3, KYSE30 and KYSE150 cells were treated with U0126 and/or Stattic, to target the STAT3 SH2 domain and prevent dimerization [30]. It was found that both pSTAT3 and pERK1/2 expression decreased upon combined treatment with U0126 and Stattic (Fig. 3A, B). U0126 had little effect on ESCC cells but acted together with Stattic to inhibit cell proliferation (Fig. 3C). The combination of trametinib, another inhibitor of MEK, and JAK1/2 inhibitor ruxolitinib can effectively inhibited colony formation and the phosphorylation of ERK1/2 and STAT3 as well (Supplementary Fig. S1A–D). Consistent with our combination therapy studies, STAT3-KO cells were more sensitive to U0126 (Fig. 3D). STAT3-KO might increase the phosphorylation of ERK1/2, resulting in cell proliferation that is more dependent on the ERK1/2 signaling pathway. We also showed that ERK2- or ERK1/2-KO enhanced the sensitivity of ESCC cells to Stattic (Fig. 3E). Given the additional evidence of pERK1/2 and pSTAT3 signaling crosstalk in ESCC cells, we monitored if ERK1/2 and STAT3 signaling contributes to cell proliferation over time using flow cytometry. KYSE30 wild-type (WT) cells stably expressing blue fluorescent protein (BFP) (WT-BFP) gave rise to dominant populations when cultured with KYSE30 ERK1/2-DKO-DsRed cells (Fig. 3F) or KYSE30 STAT3-KO-DsRed cells (Fig. 3G) treated with Stattic and trametinib, respectively. These observations indicate that the combined inhibition of dual signaling pathways suppresses cell proliferation.

### MEKi downregulates expression of the SOCS3 suppressor, resulting in activation of the STAT3 pathway

**Fig. 4:**
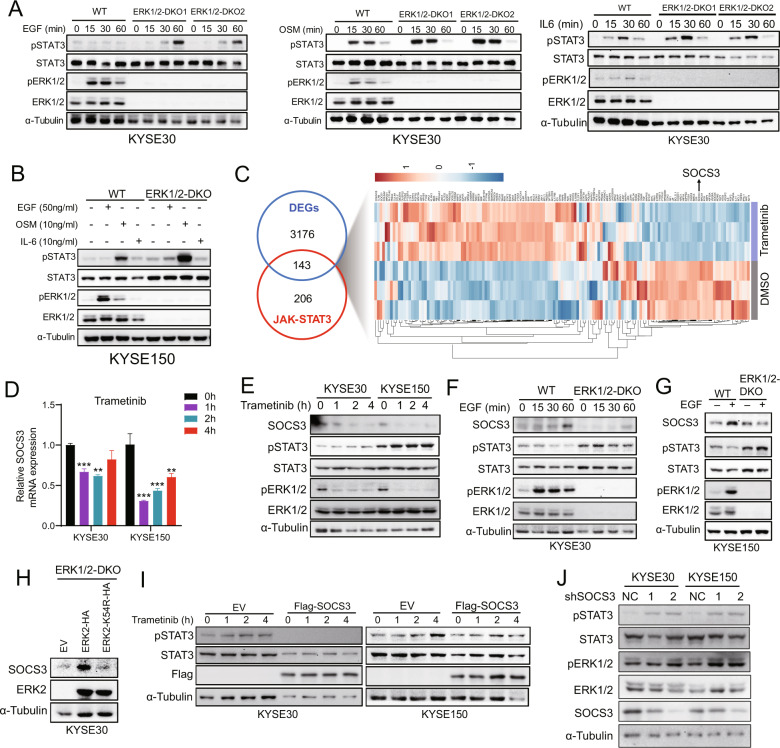
MEKi results in STAT3 phosphorylation, which occurs through SOCS3 downregulation. **A** Western blotting shows the expression of pSTAT3 and pERK in KYSE30 WT, ERK1/2-DKO-1, and ERK1/2-DKO-2 after EGF (50 ng/ml), OSM (10 ng/ml), or IL-6 (10 ng/ml) treatment. **B** KYSE150 WT and ERK1/2-DKO cells were treated with EGF (50 ng/ml), OSM (10 ng/ml), or IL-6 (10 ng/ml) for 1 h. Expressions of pSTAT3 and pERK were determined using western blotting. **C** RNA-seq heat map analyses of differentially expressed genes (DEGs) involved in the JAK-STAT3 signaling pathway after trametinib or DMSO treatment. A fold change cutoff of log2 < −0.58 or >0.58 and a *p* value cutoff of *P* < 0.05 were selected and overlapped for JAK-STAT3 signaling pathway genes. **D** qRT-PCR results show the transcription level of *SOCS3* mRNA after trametinib treatment at various time points. Error bars represent the mean ± SD. **P < 0.01, ***P < 0.001. **E** KYSE30 and KYSE150 cells were incubated with trametinib (1 μM) for 0, 1, 2, 4 h, immunoblotted for SOCS3 expression. **F**, **G** Western blotting results show the expression of SOCS3 after EGF (50 ng/ml) treatment in WT, ERK-DKO KYSE30 (**F**) and KYSE150 (**G**) cells. **H** KYSE30 ERK1/2-DKO cells stably expressing EV, ERK2-HA, and ERK2-K54R-HA were used for western blotting to determine SOCS3 expression. **I** Western blotting results show the expression of pSTAT3 and Flag-SOCS3 in KYSE30 and KYSE150 cells transduced with EV or Flag-SOCS3. Trametinib (1 μM) treatment was applied at various time points. **J** Western blotting results show the expression of pSTAT3 and pERK1/2 in KYSE30 and KYSE150 cells infected with shNC or shSOCS3 lentiviral with two target sites.

In ERK1/2-DKO KYSE30 and KYSE150 cells, expression of pSTAT3 was notably increased after EGF, oncostatin M (OSM), and interleukin-6 (IL-6) treatment when compared with WT cells (Fig. 4A, B). These results suggest that STAT3 activation due to the inhibition of the ERK signaling might occur through multiple pathways. To further explore the mechanism by which MEKi increased pSTAT3 levels, we performed RNA-seq analysis in KYSE150 cells treated with trametinib or DMSO. A total of 143 differentially expressed genes (DEGs, fold change (FC) > 1.5, *P* < 0.05) were detected, including downregulation of SOCS3 expression after trametinib treatment (Fig. 4C, Supplementary Table S2). In addition, we observed a decrease in the expression of SOCS3 at mRNA and protein levels along with an increase in pSTAT3 expression upon MEKi in ESCC cells (Fig. 4D, E). These results indicated that MEK signaling was required for SOCS3 expression. In line with this conclusion, EGF stimulated SOCS3 expression in ESCC cells, and this increase was blocked by ERK1/2-DKO (Fig. 4F, G). In addition, the expression of the ERK2 K54R kinase-inactive mutant in ERK1/2-DKO cells blocked SOCS3 expression (Fig. 4H). Flag-SOCS3 overexpression or SOCS3 knockdown resulted in a decreased or increased in STAT3 phosphorylation (Fig. 4I, J). Taken together, ERK activity increased SOCS3 expression, and suppression of *SOCS3* transcription mediated STAT3 activation in ESCC cells.

### *SOCS3* is a downstream gene of the MEK/ERK/ELK1 signaling pathway

**Fig. 5:**
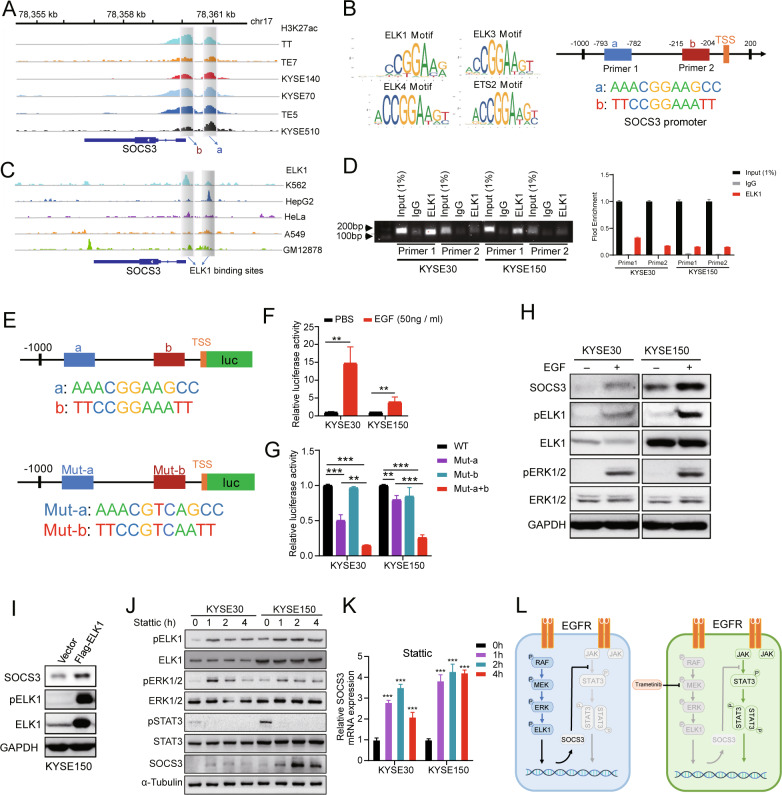
*SOCS3* is a downstream gene of the MEK/ERK/ELK1 signaling pathway. **A** ChIP-seq analyses of six ESCC cell lines revealed H3K27ac signals at the SOCS3 locus. Shadows highlighting selected constitutive promoters of SOCS3 (a and b). **B** Predicted sequences of transcription factor motifs at the *SOCS3* promoter determined using JASPAR. Predicted sequences of transcription factors ELK1, ELK3, ELK4, and ETS2. **C** ChIP-seq of ENCODE data for human malignant myeloid K562, human hepatoma HepG2, human cervical cancer HeLa, human lung cancer A549, and human B lymphocyte GM12878 cells revealed binding of ELK1 to the *SOCS3* locus. Shadows highlighting selected constitutive promoters of *SOCS3* occupied by ELK1. **D** ChIP-PCR analysis of ELK1 enrichment at the *SOCS3* promoter in KYSE30 and KYSE150 cells. Shown are representative electrophoresis (up) and qRT-PCR (down) results of two independent experiments. Error bars represent the mean ± SD. **E** Schematic diagram of luciferase reporter plasmids which were constructed containing the wild SOCS3 promoter or mutant ELK1 motif. **F** KYSE30 and KYSE150 were transfected with the WT SOCS3 promoter-reporter. After 24 h, cells were treated with EGF (50 ng/ml) for 6 h and luciferase activity was evaluated. Error bars represent the mean ± SD. **G** Luciferase activity was evaluated in mutant ELK1 motif reporter-transfected cells after 24 h. Error bars represent the mean ± SD. *P < 0.05, **P < 0.01, ***P < 0.001. **H** Western blotting results show the phosphorylation of ELK1 and the expression of SOCS3 after EGF (50 ng/ml) treatment in KYSE30 and KYSE150 cells. **I** KYSE150 cells were transfected with Flag-ELK1 and SOCS3 expression was detected via western blotting. **J** Western blotting showed the expression of activated and total ELK1, ERK1/2, STAT3 and SOCS3 expression in KYSE30 and KYSE150 treated with Stattic in a time-dependent manner. **K** qRT-PCR results show the transcription level of *SOCS3* mRNA after Stattic treatment at various time points. Error bars represent the mean ± SD. ***P < 0.001. **L** As in ESCC, EGFR signaling induced MEK/ERK activation but not STAT3 due to SOCS3 expression. MEKi caused SOCS3 downregulation and STAT3 was activated when EGFR signaling was induced.

To further characterize the activity of the *SOCS3* promoter and its associated transcription factors, we used H3K27ac ChIP-seq data of six ESCC cell lines [31]. Two putative promoter constituents were identified through the H3K27ac peaks (Fig. 5A). We further identified the binding motifs of ELK1, ELK3, ELK4, and ETS2 at the *SOCS3* promoter using the JASPAR database (Fig. 5B). ChIP-seq data of ELK1-binding sites in the Encyclopedia of DNA Elements (ENCODE) database also showed an association between ELK1 and the *SOCS3* promoter (Fig. 5C). To confirm the interaction of ELK1 and the *SOCS3* promoter, a ChIP-PCR assay was performed in KYSE30 and KYSE150 cells, and ELK1 was enriched in the a and b regions (Fig. 5D). To measure the regulatory effect of ELK1 on its target promoters, we performed a luciferase reporter assay with the *SOCS3* promoter containing either a WT or mutant ELK1-binding motif (Fig. 5E). Induction of luciferase activity, by EGF treatment, was much greater when the luciferase reporter was driven by the WT *SOCS3* promoter than when driven by the double-mutant ELK1 motif *SOCS3* promoter (Fig. 5F, G). In addition, SOCS3 expression and ELK1 phosphorylation increased upon EGF treatment and ELK1 overexpression (Fig. 5H, I). Moreover, inhibition of STAT3 signaling pathway alone induced the activation of the ERK/ELK1/SOCS3 axis in both KYSE30 and KYSE150 cells (Fig. 5J, K). These results suggest that ELK1 is phosphorylated through ERK signaling and assembles at the *SOCS3* promoter to activate the transcription of SOCS3. Thus, while EGF stimulation activates the EGFR to promote the MEK/ERK-driven transcription in ESCC, MEKi decreases SOCS3 expression and activates STAT3-dependent transcription.

### Combined trametinib and Stattic treatment induces cell cycle arrest and senescence in ESCC cells

**Fig. 6:**
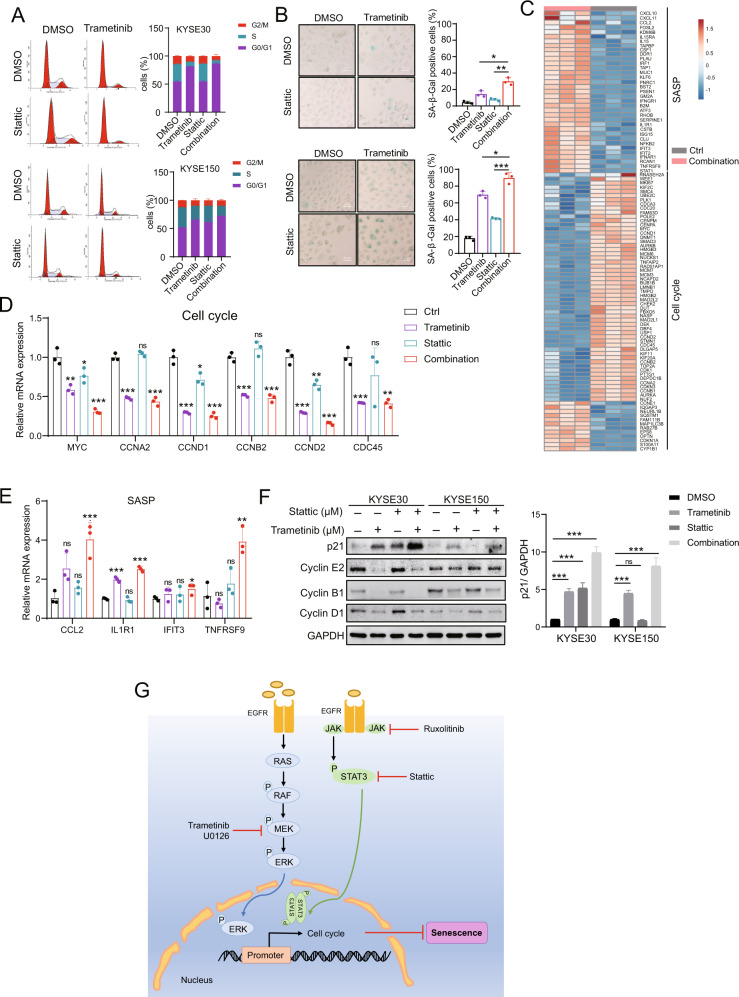
Combined trametinib and Stattic treatment induces tumor cell cycle arrest and senescence in ESCC. **A** KYSE30 and KYSE150 cells were treated with trametinib and/or Stattic for 48 h, and cell cycle analysis was performed using flow cytometry. The histogram shows the percentage of cells in different cell cycle phases. **B** KYSE30 and KYSE150 cells were treated with trametinib and/or Stattic for 48 h, and cell senescence assays were performed using SA-β-Gal staining. The histogram shows the percentage of SA-β-Gal-positive cells. Scale bar, 20 μm. Error bars represent the mean ± SD. *P < 0.05, **P < 0.01, ***P < 0.001. **C** Heat map of SASP and cell cycle-related gene expression in KYSE150 cells treated with trametinib (100 nM) and Stattic (100 nM) for 2 days. Three biological replicates are shown. **D**, **E** qRT-PCR verified the mRNA expression of the senescence-associated cell cycle (**D**) and SASP (**E**) genes. Error bars represent the mean ± SD of three experimental replicates. *P < 0.05, **P < 0.01, ***P < 0.001. **F** KYSE30 and KYSE150 cells were treated with trametinib and/or Stattic for 48 h, expressions of p21, cyclin A2, cyclin B1, and cyclin D1 were examined. Densitometry analyses of p21 represent three independent experiments. Error bars represent the mean ± SD. *P < 0.05, **P < 0.01, ***P < 0.001. **G** A schematic diagram of how MEKi and STAT3i induce cellular senescence in ESCC.

To determine the mechanism by which MEK and STAT3 dual inhibition reduced colony formation and cell proliferation, we performed cell cycle assays. Compared to either trametinib or Stattic alone, we found an increase in G1 phase arrest in cells treated with trametinib and Stattic together (Fig. 6A). We further explored how the combined inhibition affected cell senescence. As shown in Fig. 6B, the number of senescence-associated SA-β-gal-positive cells was significantly increased in the combined treatment group when compared to either trametinib or Stattic treatment alone. RNA-seq data of KYSE150 cells after inhibitor treatment revealed that the trametinib and Stattic combination downregulated a greater number of cell cycle-related genes and increased *SASP* expression compared with DMSO treatment (Fig. 6C, Supplementary Table S3). This analysis highlights that MEK and STAT3 signaling blockade activateds the cell senescence pathway. Then, we verified that the mRNA transcription of cell cycle- and SASP signaling pathway-related genes were changed after trametinib and Stattic treatment using qRT-PCR (Fig. 6D, E). Meanwhile, the expression of p21 was elevated when two inhibitors were used in combination (Fig. 6F). These results suggest that combined MEK and STAT3i suppresses ESCC cell proliferation by inducing cell senescence.

### Suppression of MEK in combination with ruxolitinib leads to regression of tumor growth in vivo

**Fig. 7:**
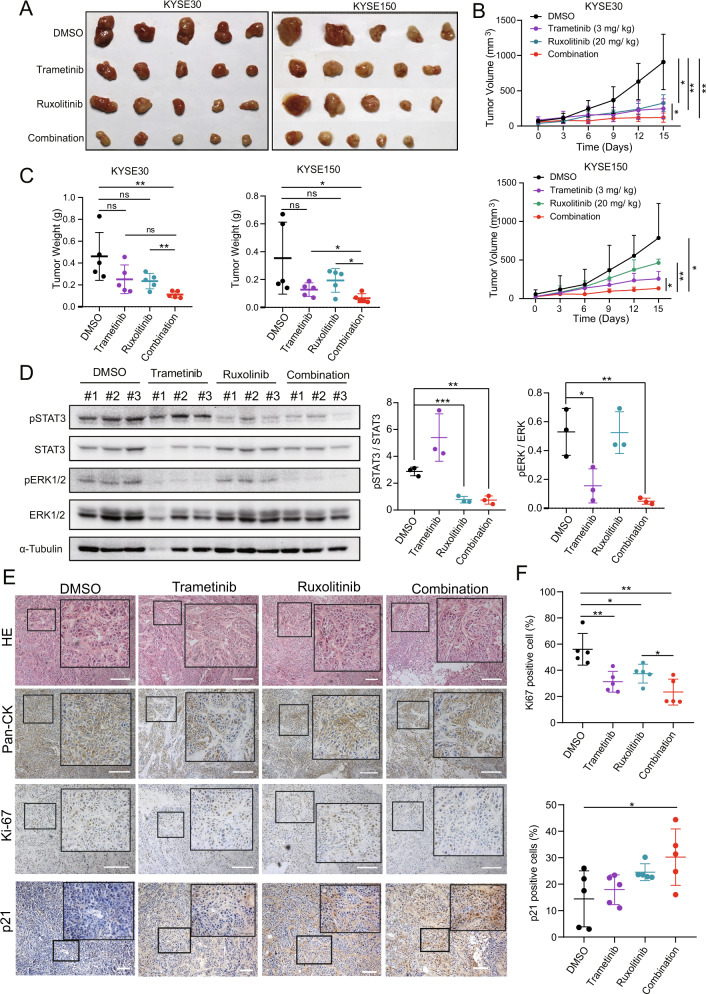
Suppression of MEK using a combination of trametinib with ruxolitinib leads to tumor growth regression in vivo. **A**–**C** Nude mice bearing KYSE30 and KYSE150 tumors were treated with the indicated inhibitors for 15 days. Tumor size (**A**), tumor volumes (**B**), and tumor mass at the endpoint (**C**) are shown. **D** Western blotting results show the expression of pSTAT3 and pERK1/2 in xenograft tumors exposed to the indicated treatment. Densitometry analyses of pSTAT3 and pERK1/2 expression normalized to STAT3 and ERK1/2 expression, respectively. Error bars represent the mean ± SD. *P < 0.05, **P < 0.01, ***P < 0.001. **E** HE staining and IHC of xenograft tumors. **F** The percentage of Ki67- and p21-positive cells were determined using IHC (right). Scale bar, 100 μm. Error bars represent the mean ± SD. *P < 0.05, **P < 0.01, ***P < 0.001.

To further identify whether the induction of senescence by MEKi and STAT3i suppresses tumor growth in vivo, KYSE30 and KYSE150 cells were implanted subcutaneously into BALB/C nude mice and treated with DMSO, trametinib, ruxolitinib, or their combination via intraperitoneal injection every 3 days. The xenografts treated with both trametinib and ruxolitinib showed significantly suppressed growth compared to those treated with trametinib or ruxolitinib alone (Fig. 7A–C). The expressions of pSTAT3 and pERK1/2 were decreased upon combination therapy compared with those with monotreatment (Fig. 7D). IHC also showed decreased Ki67 and increased p21 expression in xenografts upon combination treatment compared to the control (Fig. 7E, F). All data suggest that MEKi and STAT3i combination therapy effectively improves anti-ESCC tumor efficacy.

## Discussion

Here, we identified that the ERK signaling pathway is a potential target for ESCC therapy. ESCC cells become resistant to ERK signaling inhibition by inducing STAT3 activation. MEKi blocks the MEK/ERK/ELK1-driven transcription, decreases downstream target gene SOCS3 expression and results in the activation of STAT3 in ESCC. Combined strategy of MEKi and STAT3i can inhibit ESCC cell proliferation and induce cell senescence in vitro and in vivo.

ESCC has a high mortality rate in China. Surgical treatment combined with chemotherapy, radiotherapy, and chemoradiotherapy is the primary clinical treatment for ESCC. However, conventional cytotoxic treatments have limitations and severe adverse effects. Although the genomic features of ESCC have been well studied, few therapeutic targets and drugs are available for ESCC targeted therapy [5, 10].

*EGFR* and *ERBB2* amplification are found in the majority of ESCC patients [9]. The EGFR inhibitors gefitinib and erlotinib can effectively inhibit tyrosine kinase activity and lead to downregulation of the PI3K/AKT and MEK/ERK signaling pathways. However, only 2.8% of patients with ESCC with EGFR amplification achieved a response in a phase II trial of the EGFR inhibitor gefitinib, and the EGFR expression level was positively associated with a better prognosis [32]. These results indicate that monotherapy with EGFR inhibitors has no significant clinical effect on patients with ESCC.

The MAPK pathway is the most prominent in tumors. Recently, MEK/ERK pathway inhibitors have been proposed as a new strategy for cancers with EGFR, RAS, and RAF mutations. Trametinib is the first MEK inhibitor to receive FDA approval for the treatment of melanoma with BRAF-V600E mutations [33]. However, a single agent has poor therapeutic efficacy due to the feedback activation of oncogenic pathways and alterations in metabolic functions [34–37]. MAPK signaling is involved in cell proliferation and metastasis in ESCC as well [38, 39].

Constitutive activation of STAT3 can effectively induce malignant transformation and tumor metastasis in ESCC [40]. STAT3 is activated by various cytokines, such as IL-6, LIF, TNFα, EGF, and PDGF [41]. In addition to cytokine stimulation, protein tyrosine phosphatases and SOCS3 dysregulation also result in STAT3 activation [42, 43]. Both MAPK and STAT3 signaling pathways are activated downstream of EGFR [16, 44]. We have previously identified that blocking STAT3 signaling using STAT3β, a splice variant of STAT3 can enhance chemotherapy and chemoradiotherapy sensitivity in ESCC [45–47]. Here, we observed a negative correlation between pERK and pSTAT3 expression in ESCC cell lines (Fig. 2D). MEKi resulted in an increase in pSTAT3 after 2 hours treatment, which suggests that this compensatory activation is probably engaged through a transcriptional mechanism. RNA-seq and ChIP-seq analysis revealed that MEKi blocked SOCS3 expression, indicating that ELK1 might be a transcription factor for SOCS3 expression (Fig. 5L). The inverse correlation between MEK and STAT3 signaling and their crosstalk might serve as a negative feedback mechanism that prevents excessive cell signal activation. Consistent with these findings, Lee et al. observed that MEKi leads to autocrine activation of STAT3 via FGFR and IL-6 receptors in mutationally activated EGFR-driven non-small-cell lung cancers [37]. STAT3 inhibition (STAT3i) resulting in ERK activation may be involved in other mechanisms in ESCC and has been studied in pancreatic cancer [34]. Recently, Nagaraj et al. showed an inverse correlation between pSTAT3 and pERK1/2 in pancreatic cancer, in which STAT3i results in ERK activation through the TACE-AREG-EGFR axis [34]. These studies suggest that the intracellular complexity of signaling pathway crosstalk allows cell-dependent regulation and tumor cell develop different resistance mechanisms that drive a feedback loop for STAT3 activation in response to stress. While the crosstalk between STAT3 and ERK signaling is complex, we sought to identify its role in mediating tumor cell target therapy in our ESCC models after combined inhibition. Indeed, both STAT3 and MEKi resulted in decreased cell proliferation not only through inhibition of the STAT3 and ERK signaling feedback but also by inducing senescence via increasing cell cycle arrest and SASP (Fig. 6). Furthermore, gene KO studies indicate that downregulation of ERK1/2 or STAT3 enhances STAT3i or MEKi therapeutic efficacy in ESCC cells. Importantly, combination therapy can also play a role in tumor inhibition in vivo in ESCC xenografts. Numerous studies have shown that targeting MEK alone fails to sustain the signaling blockade [19, 21]. The identification of MEKi compensatory signaling is favorable for combination therapy to overcome the acquired resistance against a single therapeutic agent.

Senescence is a complicated physiological process that occurs in response to external and internal stimuli. The effect of inhibitor-induced tumor cell senescence has made it an attractive therapeutic strategy. Therapy-induced cellular senescence suppresses tumor growth and enhances anti-tumor immunotherapy by inducing cell cycle arrest and SASP [26, 48]. Inhibition of STAT3 activation reprograms the SASP and improves the efficacy of docetaxel-induced senescence by activating immunosurveillance in PTEN^−/−^ prostate tumors [49]. Blockade of long-term ERK signaling also induces senescence through MYC degradation and p16 reactivation in KRAS-mutant pancreatic cancer [28]. It was also previously reported that the inhibition of MAPK and CDK4/6 induces cell cycle arrest and senescence in KRAS-mutant lung cancer cells [26]. However, senescence induced by combined inhibition of STAT3 and ERK signaling has not been studied in ESCC. In this study, dual blockade of STAT3 and ERK significantly induced cell cycle arrest and increased SA-β-gal staining, as well as SASP (Fig. 6). Senescence is considered a double-edged sword in tumor development [50]. Oncogene activation that induces cell senescence during tumor initiation can inhibit tumor growth. For example, HRAS, EGFR, and HER2 activation can drive a permanent cell cycle arrest and senescence in mammary epithelial cells [51–53]. SASP can suppress tumor growth by inducing paracrine senescence and recruiting immune cells due to the release of chemokines and cytokines [54]. However, SASP also promotes tumor cell proliferation, relapse after chemotherapy, and immunosuppression [55–58]. Our data indicate that the combined inhibition of STAT3 and MEK induces ESCC cell senescence, which plays an anti-tumor role in nude mice. Whether senescence and SASP can modify the tumor microenvironment and promote anti-tumor activity requires further research.

In summary, we explored the increased phosphorylation of ERK1/2 and STAT3 in association with poor prognosis to demonstrate their potential role in ESCC targeted therapy. We further identified that MEKi induces the activation of STAT3 signaling through the ERK-ELK1-SOSC3 axis. Dual inhibition of MEK and STAT3 signaling results in the disruption of potential crosstalk disruption, which can effectively inhibit tumor growth in vitro and in vivo. These findings improve our understanding of the crosstalk between the ERK and STAT3 signaling pathways, and combination therapy with MEK and STAT3 inhibitors may be beneficial for ESCC therapy.

## Materials and methods

### Reagents and antibodies

The MEK1/2 inhibitor U0126 (V1121) was purchased from Promega. Trametinib (MEK1/2 inhibitor, HY-10999), Stattic (STAT3 inhibitor, HY-13818), and ruxolitinib (JAK1/2 inhibitor, HY-50856) were obtained from MedChemExpress (MCE, China). Blasticidin (BSD, R21001) and EGF (PHG0311) were obtained from Thermo Fisher Scientific. OSM (10452-HNAH) and human recombinant IL-6 (10395-HNAE) were purchased from Sino Biological Inc. (Beijing, China). X-gal (0428-1 G) was purchased from Maygene Inc. (Guangzhou, China).

Antibodies against STAT3 (9139 s), p-STAT3 Y705 (9145 s), p-p44/42 MAPK (ERK1/2) (4370 s), p44/42 MAPK (ERK1/2) (4695 s), cyclin E2 (4656), and cyclin B1 (12231) were obtained from Cell Signaling Technology (Shanghai, China). pERK (E-4) (sc-7383) and p-ELK-1 (B-4) (sc-8406) were purchased from Santa Cruz Biotechnology (Dallas, TX, USA). Antibodies against GAPDH (60004-1-Ig), α-tubulin (66031-1-Ig), Flag (DDDDK tag) (20543-1-AP), SOCS3 (14025-1-AP), p21 (10355-1-AP), and cyclin D1 (60186-1-Ig) were purchased from Proteintech. Anti-ELK1 antibody (ab32106) was purchased from Abcam (Shanghai, China).

### Cell culture

Sources of ESCC cell lines have been described previously [59]. The human ESCC cell lines KYSE30, KYSE150, KYSE450, KYSE510, and TE3 were grown in RPMI 1640 medium (Thermo Fisher), and TE1 and HEK293T cells were maintained in DMEM (Thermo Fisher). The media contained 10% fetal bovine serum (Thermo Fisher), penicillin (100 U/ml), and streptomycin (100 g/mL), and cells were incubated at 37 °C in 5% CO_2_. All cell lines were verified by STR analysis and free of mycoplasma contamination (IGEbio, Guangzhou, China).

### ESCC tissue specimens

ESCC tissue specimens (*n* = 301) were collected after surgical resection at the Shantou Central Hospital (Shantou, China), between 2007 and 2013, with the approval of the ethical committee of the Shantou University Medical College (SUMC-2017-12, 2018.01.01). Written informed consent was obtained from all patients. All tumors were confirmed as ESCC by pathologists in the Clinical Pathology Department of the Hospital. Only the follow-up data of patients who died from ESCC were included in tumor-related deaths, and the follow-up deadline was July 2019. Patients suffering from severe postoperative complications, other tumors, or those who died of other causes were excluded. Information on the various clinicopathological characteristics is listed in Supplementary Table S4.

### Hematoxylin and eosin (HE) staining and immunohistochemistry (IHC)

IHC of esophageal carcinoma tissues was performed as described in our previous study [60]. Anti-pERK and pSTAT3 antibodies were used. Xenograft tumors from nude mice were fixed in 4% paraformaldehyde for 6 h at room temperature and dehydrated overnight. Fixed tissues were sectioned at 4 μm and processed for HE, and IHC was performed using anti-pERK (1:500) and anti-pSTAT3 (1:200) antibodies following standard procedures [60].

### Risk score calculations and survival analysis

The expression of pERK and pSTAT3 in ESCC specimens was calculated based on the intensity of staining in tumor cells using the Vectra automated multispectral histopathological quantitative analysis system (InForm Version 2.1; PerkinElmer), and the scores (from 0 to 300) were used for the following analyses. A high or low concentration of a given protein was defined using X-tile software (Release 3.6.1). Clinical survival analyses were performed using SPSS (version 22.0; IBM, Chicago, IL, USA) or GraphPad Prism 8 software (La Jolla, CA, USA). Overall survival was defined as the time from the date of primary surgery to the date of death due to EC, and data for survivors was recorded at the last follow-up.

### RNA extraction and quantitative real-time PCR (qRT-PCR)

RNA was extracted using TRIzol (15596018, Life Technologies) as previously described [47]. Total RNA was reverse transcribed using HiScript^®^ III RT SuperMix for qPCR (+gDNA wiper) (R323-01, Vazyme) according to the manufacturer’s instructions. Quantitative PCR was performed using ChamQ Universal SYBR qPCR Master Mix (Q711-02, Vazyme) and Applied Biosystems 7500/7500 Fast Real-Time PCR System (Thermo Fisher). The primer sequences used for quantitative PCR are listed in Supplementary Table S5. *ACTB* was used as an internal reference and for normalization. mRNA expression was determined using a comparative threshold (CT) value. The CT value was normalized using the formula: ΔCT = CT (target gene) – CT (*ACTB*). Relative mRNA expression was normalized against the relative value obtained from the control group using the formula: ΔΔCT = ΔCT (treatment group) – ΔCT (control group). The expression FC was determined according to the following formula: FC = 2^−ΔΔCT^. All experiments were performed in triplicates.

### RNA-seq

RNA-seq was performed using a BGISEQ-500 system (BGI, Wuhan, China). Data were aligned using STAR (version 2.7.6a), and differentially expressed mRNAs were identified using DESeq2 (version 1.16.1). An FC cutoff of log2 < −0.58 or >0.58 and a *p* value cutoff of *P* < 0.05, were deemed significant for the regulated gene sets. JAK-STAT3 signaling pathway-related genes were retrieved from GeneCards (https://www.genecards.org/Search/Keyword?queryString=jak-STAT3).

### Chromatin immunoprecipitation sequencing (ChIP-seq) data analysis

ChIP-seq data were obtained from our previous studies using the GEO database (GEO ID: GSE76861 and GSE106563) [61, 62]. H3K27ac ChIP-seq bigwig files were obtained as previously described [31]. ELK1 ChIP-seq bigwig files in K562, HepG2, HeLa, A549, and GM12878 cell lines were obtained from the ENCODE [63]. All files were visualized using the INTEGRATIVE GENOMICS VIEWER (http://www.broadinstitute.org/igv/home). H3K27ac and ELK1 peaks within ±1 kb of the SOCS3 transcription start sites were recorded.

### ChIP-PCR assay

ChIP-PCR assays were performed as previously described [61]. In brief, 1 × 10^7^ KYSE30 and KYSE150 cells were crosslinked with formaldehyde (1%) (28908, Thermo Fisher) and neutralized with glycine. Cells were lysed, and DNA was disrupted by sonication (Covaris E220, Woburn, MA, USA). Each ultrasonic product was divided into two equal volumes. Anti-ELK1 and normal IgG were added to each volume and incubated at 4 °C overnight. Dynabeads protein A/G magnetic beads (Invitrogen) were added for 4 h at 4 °C. Complexes were immunoprecipitated, and DNA was eluted and purified using QIAquick PCR Purification Kit (28106, QIAGEN, Germany). The immunoprecipitated DNA was quantified via PCR with SOCS3 promoter-specific primers and separated on a 1.8% agarose gel. The primer sequences used for ChIP-PCR are listed in Supplementary Table S5. The relative enrichment was normalized to a 1% input. IgG antibody was used as a negative control.

### Xenograft studies

All animal studies were conducted in accordance with the protocols approved by the Animal Research Committee of the Shantou Administration Center (SUMC2021-348). Six-week-old female nude mice were randomly divided into eight groups (five mice each) and 1 × 10^6^ KYSE30 and KYSE150 cells (resuspended in 100 μL PBS) were implanted subcutaneously into the right flanks of female nude mice (Vital River Laboratories, Beijing, China). The mice were then examined every 3 days for weight and tumor growth. Ten days after cell injection when the xenograft tumors were palpable, trametinib (3 mg/kg), ruxolitinib (20 mg/kg), or both were injected intraperitoneally every 3 days to optimize the inhibitor dose in nude mice as previously reported [18, 37]. Tumor size was measured every 3 days and the volume was calculated using the following formula: (length × width^2^)/2. Fifteen days after tumor cell injection (xenograft tumor volume reached 1000 mm^3^) and the mice were euthanized. Tumors were resected and weighted, and then fixed in 4% paraformaldehyde, snap-frozen, and stored at −80 °C for further histopathological processing.

### Statistical analysis

Statistical analysis was performed using SPSS (version 17.0; SPSS Inc., Chicago, IL, USA) and GraphPad Prism 8 software (GraphPad Prism Software Inc., San Diego, CA, USA). Student’s *t* test was used for independent sample analysis. Pearson correlation analysis and U0126 IC50 values were calculated using Prism 8 software. All data represent at least three independent experiments. Overall survival curves were estimated using the Kaplan–Meier method and compared using the log-rank test. Statistical significance was set at *P* < 0.05 and denoted as *P < 0.05, **P < 0.01, ***P < 0.001.

## Supplementary information

Supplementary material

A reproducibility checklist

Original Data File

## Data Availability

All data generated or analyzed in this study are included in this paper and can be obtained from the corresponding author according to formal requirement.
