# Supplementary material for: Blocking STAT3 signaling augments MEK/ERK inhibitor efficacy in esophageal squamous cell carcinoma
Source: Cell Death Dis. 2022 May 25;13(5):496. doi: 10.1038/s41419-022-04941-3 (PMC9132929; doi:10.1038/s41419-022-04941-3)
Supplement: Supplementary file 1 — Supplementary material [file 41419_2022_4941_MOESM1_ESM.docx]

**Blocking STAT3 signaling augments MEK/ERK inhibitor efficacy in esophageal squamous cell carcinoma**

Zhen-Yuan Zheng^1,2,3^, Man-Yu Chu^1,2^, Wan Lin^1,2^, Ya-Qi Zheng^1,2^, Xiu-E Xu^1,2^, Yang Chen^1,2^, Lian-Di Liao^1,2^, Zhi-Yong Wu^4^, Shao-Hong Wang^4^, En-Min Li^1,3*^, Li-Yan Xu^1,2,3*^

^1^ The Key Laboratory of Molecular Biology for High Cancer Incidence Coastal Chaoshan Area, Department of Biochemistry and Molecular Biology, Shantou University Medical College, Shantou 515041, Guangdong, China

^2^ Guangdong Provincial Key Laboratory of Infectious Diseases and Molecular Immunopathology, Institute of Oncologic Pathology, Shantou University Medical College, Shantou University Medical College, Shantou 515041, Guangdong, China

^3^ Guangdong Esophageal Cancer Research Institute, Shantou Sub-center, Cancer Research Center, Shantou University Medical College, Shantou 515041, Guangdong, China

^4^ Shantou Central Hospital, Affiliated Shantou Hospital of Sun Yat-Sen University, Shantou 515041, Guangdong, China

***Corresponding author:*** Dr. Li-Yan Xu, Institute of Oncologic Pathology, Shantou University Medical College, No. 22, Xinling Road, Shantou 515041, Guangdong, P.R. China. Phone: 86-754-88900464; Fax: 86-754-88900847; E-mail: lyxu@stu.edu.cn.; Dr. En-Min Li, Department of Biochemistry and Molecular Biology, Shantou University Medical College, No. 22, Xinling Road, Shantou 515041, Guangdong, P.R. China. Phone: 86-754-88900413; Fax: 86-754-88900847; E-mail: nmli@stu.edu.cn.

**Description: This file contains Supplementary Figures, Supplementary Materials and Methods, and Supplementary Tables.**

**Supplementary Fig. S1 Effects of the combination of MEK/ERK and JAK/STAT3 signaling inhibitors on the growth of ESCC cells.**


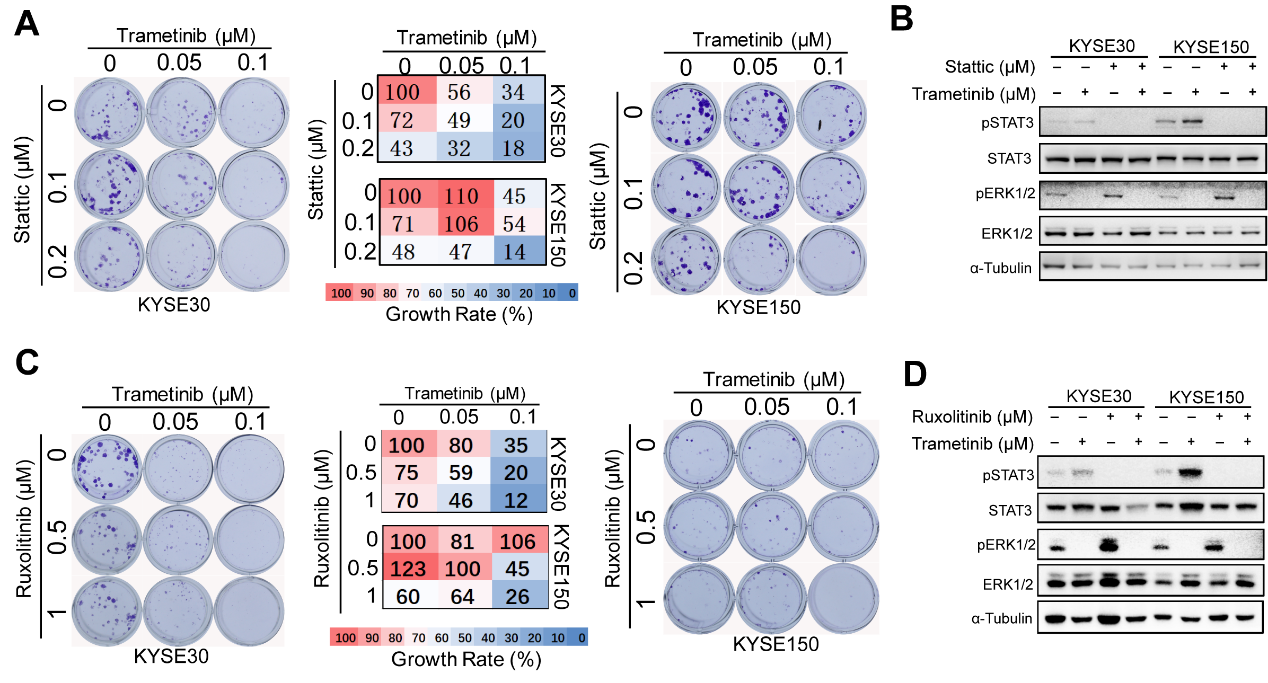


**(A) Colony formation assays of KYSE30 and KYSE150 cells treated with a combination of trametinib and Stattic at different concentrations as indicated. Percentage growth at each concentration of the inhibitor is presented (middle). (B) Western blotting results show the expressions of pSTAT3 and pERK in KYSE30 and KYSE150 cells after treatment with trametinib (1 μM) or Stattic (1 μM) for 4 h. (C) Colony formation assays of KYSE30 and KYSE150 cells treated with a combination of trametinib and ruxolitinib at different concentrations as indicated. Percentage growth at each concentration of the inhibitor is presented (middle). (D) Western blotting results show the expressions of pSTAT3 and pERK in KYSE30 and KYSE150 after treatment with trametinib (1 μM), ruxolinib (1 μM), or both for 4 h. Data are presented as the mean of three independent experiments.**

**Supplementary Fig. S2**

**
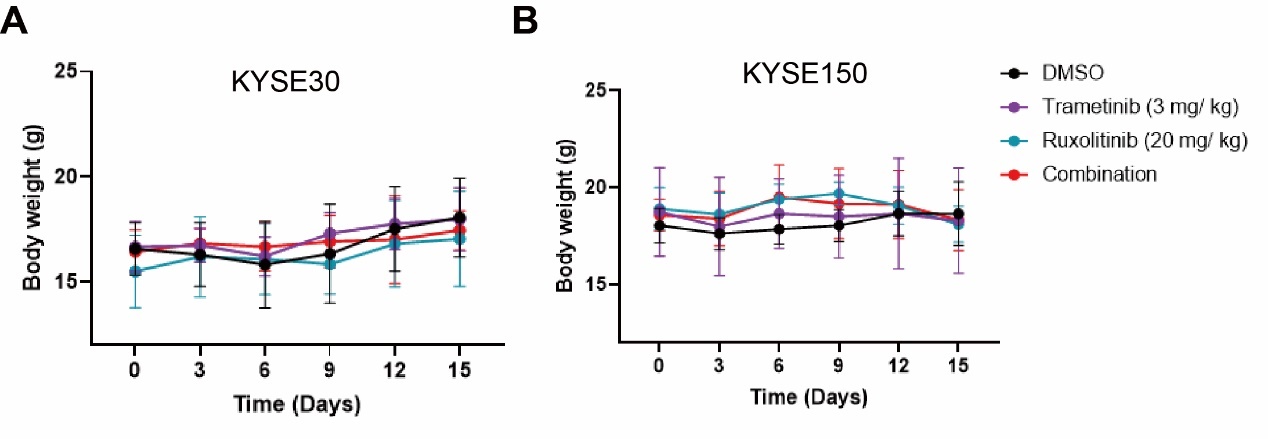
**

**(A-B)** **Body weight of nude mice implanted subcutaneously with KSYE30 (A) and KYSE150 (B) cells.** Day 0 was defined as the day of the first drug treatment, and body weight was measured every 3 days.

**Supplementary Materials and Methods**

**Plasmids, transfection, and infection**

cDNA encoding human ERK2, suppressor of cytokine signaling 3 (SOCS3), and ETS transcription factor ELK1 were amplified from reverse-transcribed cDNAs of ESCC cell lines. ERK2 was cloned into a pBOBi C-HA plasmid vector. SOCS3 and ELK1 were cloned into a pLVX plasmid vector containing a Flag tag fused to the N-terminal of the protein. The ERK2-K54R mutant was generated using the Mut Express II Fast Mutagenesis Kit V2 (C214-01, Vazyme Biotech Co., Ltd). CRISPR-Cas9 knockout cell lines were generated as previously described [2]. The gRNA sequences for ERK1, ERK2, and STAT3 were 5’-CGACCCCCTCGGTTCTACG-3’, 5’-GGCGGGCAGGT GTTCGACG-3’, and 5’-CAGCTTGACACACGGTACC-3’, respectively. The SOCS3 promoter region was amplified from the KYSE150 genome and cloned into the pGL3-basic luciferase. For shRNA experiments, KYSE30 and KYSE150 were infected with pLKO.1-shRNA-Puro lentiviruses for 48 h, selected with 1 μg·ml^-1^ puromycin for 1 week, and the cells were applied to subsequent experiments. The ELK1 motif-mutated SOCS3 promoter was constructed using the Mut Express II Fast Mutagenesis Kit V2. The constructed plasmids and vector information are shown in **Supplementary Table S6.** For plasmid transfection, Lipofectamine^TM^ 3000 transfection reagent (L3000015, Life Technologies) was used according to the manufacturer’s instructions. Lentiviral particles were generated via co-transfection with psPAX2, pMD2.G, pBOBi, or pLVX in HEK293T cells. Culture media of HEK293T cells were harvested 48 and 72 h post-transfection and used to infect KYSE30 and KYSE150 cells containing 8 μg/ml polybrene. To generate KYSE30 cells expressing BFP and DsRed, parental KYSE30 WT and STAT3-KO or ERK1/2-DKO cells were infected with plasmids carrying BFP- and DsRed-encoding genes using lentiviral and followed by single-cell cloning. Cells stably expressing BFP or DsRed were selected from 96 well plates.

**Western blotting**

Samples were prepared from cells with 1× Laemmli sample buffer (1610747, Bio-Rad, USA). The experimental procedures were performed according to the standard methods [3]. Proteins were resolved using SDS-PAGE and transferred onto polyvinylidene fluoride membranes. The membranes were blocked in 5% non-fat dry milk in Tris-buffered saline with 0.1% Tween-20 and incubated with primary antibodies at 4 °C overnight. Then, the membranes were incubated with horseradish peroxidase-conjugated secondary antibodies (1:5000) at room temperature for 1 h. Proteins were detected via chemiluminescence (sc-2048, Santa Cruz) in Image Lab^TM^ (Bio-Rad).

**Colony formation assays**

KYSE30 and KYSE150 cells were seeded in triplicate onto 24-well plates (200 cells per well) and incubated for 24 h in RPMI 1640 medium containing 10% FBS. Then, the cells were incubated with or without an inhibitor for 7-10 days. The medium was replaced every four days. Colonies were fixed with a mixture of methanol (66%) and acetic acid (33%) for 15 min and stained with 0.5% crystal violet for 30 min. Colony numbers were counted using an automatic counting tool with ImageJ (1.52a, NIH, USA). All experiments were performed in triplicate, and representative experiments are shown.

**Immuno****fluorescence (IF)**

KYSE30 and KYSE150 cells were seeded onto coverslips (F2006, Sigma) in 12-well plates. After treatment with U0126 for 2 h, the cells were fixed with 4% paraformaldehyde for 15 min and then washed three times with PBS. Triton X-100 (0.2%) was added for 10 min to allow permeation and cells were washed thrice with PBS once more. Normal donkey serum (5%, 017-000-121, Jackson) was added for 1 h at room temperature (RT). The cells were incubated with the anti-STAT3 antibody overnight at 4 °C. The cells were stained with Alexa Fluor® 647 AffiniPure Donkey Anti-Mouse IgG (H+L) (1:500, 715-605-150, Jackson) secondary antibodies for 60 min at RT and counterstained with DAPI (#D9564-10MG, Sigma) at RT for 10 min. Images were obtained and processed using laser scanning confocal microscopy (LSM800, Carl Zeiss). Co-location was analyzed using Zen Blue software (Zeiss).

**Flow cytometry**

For competitive growth assays, KYSE30 BFP^+^ and KYSE30 ERK1/2-DKO DsRed^+^ or STAT3-KO DsRed^+^ cells were mixed and treated with Stattic or trametinib. The percentages of BFP^+^ or DsRed^+^ cells were monitored using Cytek Aurora spectral flow cytometry (Cytek Biosciences Inc.).

Cell cycle assays were performed using the Cell Cycle and Apoptosis Analysis Kit (C1052, Beyotime). In brief, PBS-washed cells were fixed with cold 70% ethanol in PBS at 4 °C for 24 h and then washed twice with PBS. Cells were stained with propidium iodide solution containing 100 μg/mL RNase at 4 °C for 30 min and protected from light. Stained cells were analyzed using a BD Accuri C6 (BD Biosciences, USA). The results were processed using ModFit LT software.

**Luciferase assay**

Sixteen cancer-related signaling pathway reporter gene vectors were purchased from Promega (**Supplementary Table S1**). Luciferase reporter genes or pGL3-basic control and Renilla luciferase plasmid were transfected into cells, and the cells were cultured for 24 h as previously described [4]. After 6–8 h of U0126 or EGF stimulation, cells were lysed and Dual-Luciferase Assay (Promega) was performed. Each experiment was independently repeated three times.

**β-Galactosidase (SA-β-gal) staining**

For cell senescence, KYSE30 and KYSE150 cells at 20% confluence were treated with 1 μM trametinib, 1 μM Stattic, or a combination of both for 48 h. SA-β-Gal staining was performed according to the provided protocol [5]. Cells were washed in PBS and fixed for 10 min at RT in 2% formaldehyde/0.2% glutaraldehyde. After washing in PBS and incubation at 37 °C in a CO_2_ incubator with a senescent-cell staining solution (40 mM citric acid/sodium phosphate, pH 6.0, 5 mM potassium ferrocyanide, 5 mM potassium ferricyanide, 150 mM NaCl, 2 mM MgCl_2_, and 1 mg/mL X-gal). After 12 h of staining, the cells were examined using a Zeiss Axio Vert A1.

**Table S****1** Sixteen cancer-related signaling pathway reporter gene vectors

| NO. | Vector | Response Element | Transcription factor | Signaling pathway |
| --- | --- | --- | --- | --- |
| 1 | pGL4.49 | TCF-LEF Response Element (TCF) | LEF-TCF | Wnt |
| 2 | pGL4.48 | Smad-binding element (SBE) | SMAD3, SMAD4 | TGF-β |
| 3 | pGL4.47 | SIS-inducible element (SIE) | STAT3, STAT3 | IL6 |
| 4 | pGL4.45 | Interferon-stimulated response element (ISRE) | STAT1, STAT2 | IFN-α |
| 5 | pGL4.44 | AP1 response element (ARE) | AP1 | MAPK/JNK |
| 6 | pGL4.43 | Xenobiotic response element (XRE) | AhR | Xenobiotic stress |
| 7 | pGL4.42 | Hypoxia response element (HRE) | HIF1α | Hypoxia |
| 8 | pGL4.41 | Heat shock response element (HSE) | HSF1 | Heat shock |
| 9 | pGL4.40 | Metal response element (MRE) | MTF1 | Heavy metal stress |
| 10 | pGL4.38 | p53 response element (p53) | P53 | DNA damage |
| 11 | pGL4.37 | Antioxidant response element (ARE) | Nrf2 | Oxidative stress |
| 12 | pGL4.34 | Serum Response Factor Response Element (SRF) | SRF | RhoA |
| 13 | pGL4.33 | Serum Response Element (SRE) | ELK-1/SRF | MAPK/ERK |
| 14 | pGL4.32 | NF-κB response element (NFκB) | NF-κB | NF-κB |
| 15 | pGL4.30 | NFAT response element (NFAT) | NFAT | Calcium/Calcineurin |
| 16 | pGL4.29 | cAMP response element (CRE) | CREB | cAMP/PKA |

| **Table S2 Differentially Expressed Genes trametinib- vs. DMSO-treated cells** | | | | | | |
| --- | --- | --- | --- | --- | --- | --- |
|  | Trametinib_1 | Trametinib_2 | Trametinib_3 | DMSO_1 | DMSO_2 | DMSO_3 |
| STAT3 | 0.00342018 | 0.0033948 | 0.003255488 | 0.002472 | 0.002549 | 0.0024619 |
| IL6 | 0.00042744 | 0.00066165 | 0.000486495 | 0.001163 | 0.001486 | 0.0010818 |
| IL6ST | 0.00176422 | 0.00175114 | 0.00164079 | 0.001672 | 0.001829 | 0.0017567 |
| SOCS1 | 0.00046355 | 0.00046429 | 0.000563771 | 0.000624 | 0.000357 | 0.0002235 |
| JAK1 | 0.00130841 | 0.0013516 | 0.001341743 | 0.000838 | 0.000895 | 0.0008893 |
| JAK2 | 0.0000963 | 0.00011012 | 0.0000994 | 7.38E-05 | 8.15E-05 | 0.0000869 |
| SOCS3 | 0.00693604 | 0.00638776 | 0.007343959 | 0.010748 | 0.010265 | 0.0107804 |
| CD274 | 0.00231952 | 0.00247159 | 0.00253978 | 0.002158 | 0.002286 | 0.0022225 |
| PTPN6 | 0.00381068 | 0.00370853 | 0.003864802 | 0.002827 | 0.002312 | 0.002823 |
| FGF2 | 0.00052597 | 0.00053763 | 0.000491846 | 0.000793 | 0.000932 | 0.0007608 |
| SRC | 0.00159613 | 0.0014493 | 0.001450236 | 0.001863 | 0.001662 | 0.0017507 |
| EGFR | 0.00552658 | 0.00558303 | 0.005270749 | 0.002908 | 0.002896 | 0.0028614 |
| MAPK1 | 0.00170456 | 0.00184404 | 0.001693049 | 0.001749 | 0.001925 | 0.0018694 |
| MIR221 | 0.00218105 | 0.00655364 | 0.002730622 | 0.001814 | 0.003208 | 0.0018399 |
| PTPN11 | 0.00279896 | 0.00287626 | 0.002856689 | 0.003405 | 0.003638 | 0.0035768 |
| OSMR | 0.00337457 | 0.00349935 | 0.003413325 | 0.003649 | 0.003719 | 0.003484 |
| IL11 | 0.00036204 | 0.00038222 | 0.000284211 | 0.000662 | 0.000576 | 0.0006273 |
| IGF1R | 0.00044652 | 0.00042877 | 0.000407662 | 0.000367 | 0.000349 | 0.0003432 |
| BRCA1 | 0.00033693 | 0.00031194 | 0.000332258 | 0.000434 | 0.000429 | 0.0004772 |
| BCL2 | 0.0000396 | 0.0000375 | 0.0000309 | 0.000076 | 6.66E-05 | 0.0000608 |
| NFKB1 | 0.00059396 | 0.00060877 | 0.000611326 | 0.000577 | 0.000553 | 0.000562 |
| TGFB1 | 0.0017697 | 0.00169631 | 0.001736088 | 0.002697 | 0.002269 | 0.0024454 |
| SAA1 | 0.00490723 | 0.005009 | 0.00453931 | 0.002149 | 0.002176 | 0.0018457 |
| KIF20A | 0.01813534 | 0.01813791 | 0.018891864 | 0.025459 | 0.025449 | 0.0272279 |
| LINC00518 | 0.0000529 | 0.0000106 | 0.0000212 | 1.17E-05 | 1.04E-05 | 0.0000476 |
| MAPK3 | 0.01390222 | 0.01397514 | 0.014190116 | 0.013643 | 0.011767 | 0.0135608 |
| DICER1 | 0.0014887 | 0.0014662 | 0.001411439 | 0.001393 | 0.001334 | 0.0014025 |
| SPTBN1 | 0.00266756 | 0.00265246 | 0.002457075 | 0.001963 | 0.001935 | 0.0020286 |
| SOCS5 | 0.00041307 | 0.00038298 | 0.000371988 | 0.000514 | 0.000539 | 0.0004654 |
| IL17RB | 0.0000711 | 0.0000681 | 0.0000681 | 0.000048 | 5.16E-05 | 0.0000661 |
| CUL4A | 0.00262794 | 0.00274733 | 0.002765458 | 0.002613 | 0.00271 | 0.0025792 |
| FGFR2 | 0.00211564 | 0.0020214 | 0.002128887 | 0.00188 | 0.001917 | 0.001915 |
| JAK3 | 0.00021945 | 0.00024536 | 0.000235135 | 0.000207 | 0.000183 | 0.0001693 |
| TNF | 0.0000215 | 0 | 0 | 2.38E-05 | 4.21E-05 | 0.0000241 |
| GABBR1 | 0.00052874 | 0.00049388 | 0.000467273 | 0.000389 | 0.00032 | 0.000349 |
| HIF1A | 0.00315502 | 0.003335 | 0.003295425 | 0.004759 | 0.005008 | 0.0046679 |
| VEGFC | 0.0000425 | 0.0000403 | 0.0000502 | 0.00012 | 0.000152 | 0.0001169 |
| ACVR1B | 0.00096589 | 0.00097268 | 0.000938577 | 0.000833 | 0.000833 | 0.0008583 |
| RNF6 | 0.0006997 | 0.00068831 | 0.000719906 | 0.000608 | 0.000696 | 0.0006583 |
| RPL34 | 0.04614775 | 0.04728574 | 0.048094842 | 0.045785 | 0.048262 | 0.0485936 |
| SLC11A2 | 0.00136481 | 0.00155709 | 0.001467455 | 0.001389 | 0.001466 | 0.001425 |
| CKS1B | 0.01185599 | 0.01211427 | 0.011956159 | 0.020001 | 0.02082 | 0.0210505 |
| RPN2 | 0.00830679 | 0.00813645 | 0.008252101 | 0.008169 | 0.008091 | 0.0083811 |
| VTCN1 | 0.00060957 | 0.0005593 | 0.000588446 | 4.89E-06 | 1.12E-05 | 0.0000149 |
| LIF | 0.0009652 | 0.00085519 | 0.000817993 | 0.003962 | 0.003941 | 0.0039042 |
| BIRC5 | 0.00543987 | 0.00540179 | 0.005708408 | 0.009699 | 0.01016 | 0.0101882 |
| LIFR | 0.00021431 | 0.00024188 | 0.000219562 | 0.000238 | 0.000256 | 0.0002656 |
| MCL1 | 0.07723977 | 0.07775365 | 0.077832268 | 0.056314 | 0.060138 | 0.0575843 |
| BCL2L1 | 0.00067383 | 0.00066691 | 0.00069589 | 0.001631 | 0.001561 | 0.0015709 |
| ERBB2 | 0.00313861 | 0.00311983 | 0.002906926 | 0.002265 | 0.001904 | 0.0021732 |
| MYC | 0.00259527 | 0.00294668 | 0.002579528 | 0.003856 | 0.003934 | 0.0036657 |
| NOTCH1 | 0.00417921 | 0.00396797 | 0.003937748 | 0.001414 | 0.00134 | 0.001307 |
| TWIST1 | 0.000059 | 0.0000413 | 0.0000548 | 8.93E-05 | 9.65E-05 | 0.0000947 |
| S1PR1 | 0.0000521 | 0.0000261 | 0.0000522 | 9.62E-06 | 0.000017 | 9.76E-06 |
| ID1 | 0.02908972 | 0.02720354 | 0.031406591 | 0.023699 | 0.022803 | 0.0276194 |
| KLRK1 | 0.0000213 | 0.0000305 | 0.0000122 | 3.38E-06 | 2.99E-06 | 3.42E-06 |
| TYK2 | 0.00294142 | 0.00299945 | 0.002977662 | 0.002776 | 0.002434 | 0.0025279 |
| PIK3R1 | 0.0003196 | 0.0003208 | 0.000279313 | 0.000224 | 0.000219 | 0.0002135 |
| LEPR | 0.0000802 | 0.0000903 | 0.0000808 | 8.53E-05 | 0.000102 | 0.0001032 |
| CCND1 | 0.01015794 | 0.00992074 | 0.010249574 | 0.025497 | 0.024851 | 0.023845 |
| CDK2 | 0.00807271 | 0.00751163 | 0.007788561 | 0.010705 | 0.011095 | 0.0107695 |
| CDKN2A | 0.01232773 | 0.01152848 | 0.011904217 | 0.008098 | 0.007094 | 0.0081052 |
| JAG1 | 0.00525354 | 0.00547341 | 0.005312649 | 0.004441 | 0.004678 | 0.0041106 |
| CASP3 | 0.0013351 | 0.00128811 | 0.001307189 | 0.001293 | 0.001285 | 0.0013086 |
| CDH1 | 0.00888033 | 0.00897265 | 0.008957923 | 0.00612 | 0.006232 | 0.0060424 |
| MMP3 | 0.00010398 | 0.00014135 | 0.000156221 | 2.47E-05 | 2.19E-05 | 0 |
| IL6R | 0.00054294 | 0.00057345 | 0.000558618 | 0.000247 | 0.000208 | 0.0002174 |
| NFE2L2 | 0.00167176 | 0.00172239 | 0.001565509 | 0.001441 | 0.001291 | 0.0012661 |
| CDKN1A | 0.00995388 | 0.01049512 | 0.010286959 | 0.002411 | 0.002272 | 0.0023535 |
| STK11 | 0.00126748 | 0.00115718 | 0.001246081 | 0.001523 | 0.001432 | 0.0013324 |
| CCL2 | 0.00026947 | 0.00035987 | 0.000569785 | 3.32E-05 | 0.000411 | 0.0000337 |
| FER | 0.00013859 | 0.00014172 | 0.000138141 | 0.000109 | 0.000108 | 0.0001032 |
| POU5F1 | 0.0000399 | 0.0000472 | 0.0000726 | 4.42E-05 | 3.56E-05 | 0.000053 |
| PLOD2 | 0.00098601 | 0.00106088 | 0.001033691 | 0.000851 | 0.000923 | 0.0008515 |
| BMX | 6.44E-07 | 0.00000129 | 0.000000645 | 0 | 0 | 0 |
| BIRC6 | 0.00077007 | 0.0007813 | 0.00073946 | 0.000703 | 0.000678 | 0.0006944 |
| S100A8 | 0.00061696 | 0.00066944 | 0.001132878 | 0.000057 | 0 | 0.0000578 |
| SRF | 0.00713228 | 0.00709122 | 0.006624541 | 0.006243 | 0.005687 | 0.0059988 |
| GADD45G | 0.00111776 | 0.00088792 | 0.000694874 | 0.000898 | 0.000794 | 0.0008671 |
| GPX8 | 0.00671012 | 0.00630991 | 0.007268647 | 0.00472 | 0.005541 | 0.0059611 |
| CTF1 | 0.00070948 | 0.00047945 | 0.000710598 | 0.000294 | 0.000176 | 0.0003173 |
| AKT1 | 0.0029422 | 0.0027733 | 0.002714612 | 0.003417 | 0.003208 | 0.0034638 |
| PIK3CA | 0.00062161 | 0.00060902 | 0.000605119 | 0.000496 | 0.000535 | 0.0005175 |
| MDM2 | 0.00097728 | 0.00103486 | 0.001072645 | 0.001276 | 0.0015 | 0.0013084 |
| ADK | 0.00032809 | 0.00033577 | 0.000340665 | 0.000296 | 0.000321 | 0.0003117 |
| FOS | 0.00015478 | 0.00032727 | 0.00015502 | 0.001468 | 0.001535 | 0.0018763 |
| ITGB1 | 0.0041479 | 0.00427815 | 0.004247575 | 0.005091 | 0.005135 | 0.005101 |
| MAPK8 | 0.00051319 | 0.00047634 | 0.000513103 | 0.000532 | 0.00061 | 0.0005586 |
| SOD2 | 0.00195487 | 0.00202055 | 0.00200096 | 0.001584 | 0.001771 | 0.0016324 |
| PIM1 | 0.00735098 | 0.00754511 | 0.007886862 | 0.010877 | 0.010646 | 0.0097403 |
| CREB1 | 0.00073149 | 0.00072539 | 0.00074153 | 0.000608 | 0.000681 | 0.0006622 |
| JUN | 0.01593744 | 0.01732545 | 0.016551322 | 0.006648 | 0.006357 | 0.0072348 |
| NF1 | 0.00076199 | 0.00075782 | 0.000720286 | 0.000996 | 0.000983 | 0.0009802 |
| ESR2 | 0.0000124 | 0.0000131 | 0.0000127 | 3.89E-06 | 4.82E-06 | 3.69E-06 |
| WNT5A | 0.000067 | 0.0000504 | 0.0000743 | 2.65E-06 | 7.04E-06 | 0.0000108 |
| TNFRSF1A | 0.00831636 | 0.00858371 | 0.008262642 | 0.008615 | 0.007226 | 0.0078778 |
| SMARCA2 | 0.00023541 | 0.00021933 | 0.000215694 | 0.000158 | 0.000172 | 0.0001771 |
| RORA | 9.62E-07 | 0.00000145 | 0.0000012 | 8.89E-07 | 1.49E-06 | 0.0000018 |
| RPS6KB1 | 0.00071712 | 0.00072344 | 0.000735841 | 0.000898 | 0.000952 | 0.0009542 |
| RAC1 | 0.01790639 | 0.01780773 | 0.017407176 | 0.015829 | 0.015025 | 0.015427 |
| NRAS | 0.01602917 | 0.01629918 | 0.016375471 | 0.014025 | 0.015461 | 0.0148821 |
| VEGFA | 0.00068901 | 0.00074489 | 0.000748522 | 0.002478 | 0.002452 | 0.0029114 |
| YES1 | 0.00107814 | 0.00108052 | 0.001081808 | 0.001151 | 0.001176 | 0.0011216 |
| PTGS2 | 0.00036475 | 0.00033776 | 0.000454935 | 0.000275 | 0.00025 | 0.0002167 |
| RB1 | 0.00029565 | 0.00030478 | 0.000286858 | 0.000249 | 0.000266 | 0.0002564 |
| IRAK4 | 0.00082355 | 0.00086573 | 0.000797618 | 0.00037 | 0.00043 | 0.0004304 |
| GFAP | 0.0000129 | 0.00000864 | 0 | 1.44E-05 | 0 | 0.0000291 |
| CDKN1B | 0.00875292 | 0.00846575 | 0.008229505 | 0.008598 | 0.008839 | 0.0080901 |
| YAP1 | 0.00108657 | 0.0011193 | 0.001101842 | 0.001173 | 0.001254 | 0.0011939 |
| SOX2 | 0.00274455 | 0.00353097 | 0.004075932 | 0.001023 | 0.000951 | 0.0005589 |
| PRKAA1 | 0.00640138 | 0.00640247 | 0.006213033 | 0.005596 | 0.005983 | 0.0057808 |
| PRKAB1 | 0.00274962 | 0.00281837 | 0.002899808 | 0.002242 | 0.002285 | 0.0023798 |
| GPI | 0.00691476 | 0.00692306 | 0.007226555 | 0.0104 | 0.010772 | 0.0107642 |
| NR4A3 | 0.0000132 | 0.0000145 | 0.0000238 | 0.000019 | 3.49E-05 | 0.0000163 |
| STAT5A | 0.00000731 | 0.00000488 | 0 | 0 | 0 | 2.74E-06 |
| PPARA | 0.00029451 | 0.000302 | 0.000284118 | 0.000348 | 0.000305 | 0.0003212 |
| MUC1 | 0.00469302 | 0.00543079 | 0.00514529 | 0.000836 | 0.000855 | 0.0007258 |
| NRG1 | 0.0000456 | 0.0000456 | 0.0000455 | 0.00015 | 0.00016 | 0.0001401 |
| INPP5D | 0.0000121 | 0.0000149 | 0.0000133 | 6.82E-05 | 8.23E-05 | 0.0000705 |
| ERN1 | 0.00014003 | 0.0001422 | 0.00012402 | 0.000149 | 0.000134 | 0.0001444 |
| XBP1 | 0.01218775 | 0.01214815 | 0.012640495 | 0.016242 | 0.016303 | 0.01684 |
| PITX1 | 0.00623055 | 0.00634145 | 0.006450642 | 0.006611 | 0.005698 | 0.0061203 |
| PML | 0.00274022 | 0.0024455 | 0.002425284 | 0.001941 | 0.001815 | 0.0018355 |
| PDIA3 | 0.01476614 | 0.01470344 | 0.015358454 | 0.01368 | 0.015571 | 0.0143364 |
| ADIPOR1 | 0.00697841 | 0.00733504 | 0.007422081 | 0.007542 | 0.007146 | 0.0072015 |
| IL10RB | 0.0000244 | 0.0000272 | 0.0000394 | 0.000015 | 1.99E-05 | 0.0000214 |
| TLR9 | 0.00000585 | 0 | 0 | 6.5E-06 | 0 | 0 |
| PRDM1 | 0.00000503 | 0.00000756 | 0.00000504 | 8.37E-06 | 4.94E-06 | 5.66E-06 |
| SNAI2 | 0.01868081 | 0.0189554 | 0.01741498 | 0.012509 | 0.011696 | 0.0118638 |
| PGRMC1 | 0.01231907 | 0.01173054 | 0.012591953 | 0.01241 | 0.012393 | 0.0130758 |
| S1PR2 | 0.00075058 | 0.00075178 | 0.000709664 | 0.000866 | 0.000954 | 0.0009388 |
| AQP9 | 0.00000124 | 0.00000125 | 0 | 0 | 0 | 0 |
| COL5A2 | 0.0000086 | 0.00000708 | 0.00000739 | 7.5E-06 | 6.63E-06 | 5.53E-06 |
| ITGA1 | 0.0000389 | 0.0000441 | 0.00004 | 0.000025 | 2.65E-05 | 0.0000238 |
| PARD3 | 0.0000991 | 0.0000965 | 0.0000947 | 0.000074 | 7.81E-05 | 0.0000757 |
| EHF | 0.00081944 | 0.00088778 | 0.000805836 | 5.94E-05 | 3.79E-05 | 0.0000468 |
| CLCF1 | 0.00055224 | 0.00053528 | 0.000493635 | 0.000428 | 0.000518 | 0.0004809 |
| PTGER1 | 0.0000821 | 0.0000617 | 0.000123331 | 4.55E-05 | 2.01E-05 | 0 |
| DCBLD2 | 0.00029282 | 0.00030173 | 0.000324241 | 0.000188 | 0.000222 | 0.0002118 |
| DUSP22 | 0.00035327 | 0.00032194 | 0.000332896 | 0.000277 | 0.000228 | 0.0002944 |
| MEST | 0.00000293 | 0 | 0.00000293 | 0 | 2.87E-06 | 6.59E-06 |
| ZC3H12A | 0.00306634 | 0.00270779 | 0.003010604 | 0.001724 | 0.001512 | 0.0014558 |
| IFNL1 | 0.0000253 | 0.0000254 | 0.0000254 | 0.00014 | 7.45E-05 | 0.0001139 |

| **Table S3 Differentially Expressed Genes between combined- vs. DMSO-treated cells** | | | | | | | |
| --- | --- | --- | --- | --- | --- | --- | --- |
|  | Gene | Combination_1 | Combination_2 | Combination_3 | DMSO_1 | DMSO_2 | DMSO_3 |
| Cell cycle | CCND1 | 0.011689 | 0.012055 | 0.011533 | 0.025497 | 0.024851 | 0.023845 |
|  | HMGB2 | 0.028615 | 0.024694 | 0.026346 | 0.049747 | 0.052626 | 0.051961 |
|  | TNFAIP2 | 0.009529 | 0.008217 | 0.009075 | 0.017446 | 0.015723 | 0.016888 |
|  | AURKB | 0.01024 | 0.009199 | 0.009724 | 0.015199 | 0.014901 | 0.016556 |
|  | MCM3 | 0.021484 | 0.020635 | 0.021171 | 0.033692 | 0.033004 | 0.034287 |
|  | POLE2 | 0.000404 | 0.000466 | 0.000371 | 0.000657 | 0.000682 | 0.000638 |
|  | CCNA2 | 0.014364 | 0.012485 | 0.012966 | 0.019713 | 0.020391 | 0.021317 |
|  | RNASEH2A | 0.000134 | 0.000103 | 0.000133 | 0.00016 | 0.000155 | 0.000255 |
|  | HMGB3 | 0.005777 | 0.005495 | 0.005598 | 0.008256 | 0.008153 | 0.009078 |
|  | TMPO | 0.006994 | 0.00678 | 0.006665 | 0.010171 | 0.010279 | 0.010352 |
|  | AURKA | 0.008248 | 0.007286 | 0.007127 | 0.010782 | 0.011536 | 0.011647 |
|  | MCM6 | 0.003618 | 0.003608 | 0.003536 | 0.005117 | 0.005174 | 0.005508 |
|  | BUB1B | 0.001383 | 0.001277 | 0.001303 | 0.001923 | 0.001907 | 0.001965 |
|  | CCNB1 | 0.029195 | 0.026659 | 0.026529 | 0.038025 | 0.041575 | 0.040551 |
|  | CDKN3 | 0.002411 | 0.002195 | 0.002246 | 0.003181 | 0.003292 | 0.003416 |
|  | CENPM | 0.002064 | 0.001894 | 0.001991 | 0.002951 | 0.00283 | 0.00266 |
|  | DUT | 0.007611 | 0.007227 | 0.00761 | 0.010188 | 0.011391 | 0.010232 |
|  | DLGAP5 | 0.001487 | 0.001423 | 0.001294 | 0.001805 | 0.001965 | 0.002151 |
|  | DEK | 0.008442 | 0.00809 | 0.008127 | 0.011223 | 0.011973 | 0.011521 |
|  | NUF2 | 0.000555 | 0.000501 | 0.000485 | 0.000692 | 0.000741 | 0.000735 |
|  | LMNB1 | 0.002885 | 0.00273 | 0.002835 | 0.003908 | 0.003868 | 0.004082 |
|  | NCAPD2 | 0.008814 | 0.008697 | 0.008733 | 0.012285 | 0.011894 | 0.012295 |
|  | CDCA3 | 0.009609 | 0.007396 | 0.008445 | 0.012125 | 0.010578 | 0.012409 |
|  | CENPA | 0.000854 | 0.000816 | 0.000913 | 0.001159 | 0.001241 | 0.001131 |
|  | CDK1 | 0.00721 | 0.006113 | 0.005951 | 0.008501 | 0.008806 | 0.008926 |
|  | DEPDC1B | 0.000599 | 0.000538 | 0.000548 | 0.000725 | 0.000765 | 0.000784 |
|  | CCNB2 | 0.006797 | 0.00573 | 0.005982 | 0.007919 | 0.008576 | 0.008437 |
|  | USP1 | 0.006146 | 0.00624 | 0.006262 | 0.007871 | 0.008694 | 0.008519 |
|  | TOP2A | 0.01267 | 0.011404 | 0.011064 | 0.015347 | 0.015726 | 0.016178 |
|  | FBXO5 | 0.003128 | 0.002804 | 0.002909 | 0.00378 | 0.004249 | 0.003819 |
|  | NASP | 0.005932 | 0.005839 | 0.005474 | 0.007386 | 0.007681 | 0.007654 |
|  | MKI67 | 0.013776 | 0.014199 | 0.013175 | 0.018307 | 0.017041 | 0.01881 |
|  | RAD51AP1 | 0.002235 | 0.002042 | 0.002185 | 0.002757 | 0.002787 | 0.0028 |
|  | KIF2C | 0.004396 | 0.003805 | 0.003759 | 0.0052 | 0.00489 | 0.005339 |
|  | DNMT1 | 0.002127 | 0.002159 | 0.002133 | 0.002777 | 0.002643 | 0.002709 |
|  | KIF11 | 0.002125 | 0.001872 | 0.001771 | 0.002342 | 0.002451 | 0.00251 |
|  | SMC4 | 0.004124 | 0.003837 | 0.003585 | 0.004933 | 0.00461 | 0.005 |
|  | CDC20 | 0.05338 | 0.045922 | 0.049989 | 0.061594 | 0.06063 | 0.065104 |
|  | STMN1 | 0.00851 | 0.008573 | 0.008738 | 0.0105 | 0.011067 | 0.010668 |
|  | FAM83D | 0.003649 | 0.003456 | 0.003808 | 0.004287 | 0.004485 | 0.004835 |
|  | UBE2C | 0.045382 | 0.037473 | 0.039564 | 0.050337 | 0.049583 | 0.0506 |
|  | KIF20A | 0.023177 | 0.020522 | 0.02001 | 0.025459 | 0.025449 | 0.027228 |
|  | WEE1 | 0.003459 | 0.003676 | 0.003538 | 0.004063 | 0.00463 | 0.004388 |
|  | PLK1 | 0.010803 | 0.010143 | 0.010967 | 0.013279 | 0.012243 | 0.013501 |
|  | NUCKS1 | 0.004548 | 0.004445 | 0.004467 | 0.005361 | 0.00546 | 0.005599 |
|  | FAM111B | 0.002217 | 0.002157 | 0.00223 | 0.001708 | 0.001847 | 0.001755 |
|  | RAB27B | 0.00023 | 0.000237 | 0.000234 | 0.000183 | 0.000197 | 0.000183 |
|  | IQGAP3 | 0.003276 | 0.002985 | 0.003076 | 0.002539 | 0.002334 | 0.002534 |
|  | NEURL1B | 0.000943 | 0.000939 | 0.000963 | 0.000781 | 0.000714 | 0.000759 |
|  | EPS8 | 0.000399 | 0.000409 | 0.000381 | 0.000312 | 0.000311 | 0.000316 |
|  | MAP1LC3B | 0.004077 | 0.003781 | 0.003831 | 0.002537 | 0.002717 | 0.002586 |
|  | SQSTM1 | 0.010287 | 0.009103 | 0.010004 | 0.006584 | 0.006393 | 0.006432 |
|  | S100A11 | 0.053485 | 0.059285 | 0.058369 | 0.035647 | 0.032841 | 0.034843 |
|  | CCNE1 | 0.000411 | 0.000383 | 0.00046 | 0.000244 | 0.000282 | 0.000215 |
|  | OPTN | 0.004641 | 0.004723 | 0.004418 | 0.002298 | 0.002152 | 0.002066 |
|  | CYP1B1 | 0.000389 | 0.00046 | 0.00041 | 0.00012 | 0.000132 | 0.000117 |
|  | CDKN1A | 0.009603 | 0.009867 | 0.010929 | 0.002411 | 0.002272 | 0.002354 |
| SASP | CXCL11 | 0.000117 | 3.07E-05 | 5.49E-05 | 8.52E-06 | 7.54E-06 | 0 |
|  | TNFRSF9 | 2.84E-05 | 2.18E-05 | 1.78E-05 | 0 | 4.27E-06 | 2.45E-06 |
|  | RHOB | 0.056882 | 0.059564 | 0.061458 | 0.008033 | 0.006932 | 0.007585 |
|  | MUC1 | 0.004822 | 0.006244 | 0.006273 | 0.000836 | 0.000855 | 0.000726 |
|  | CXCL10 | 0.000163 | 7.49E-05 | 0.000128 | 0 | 2.45E-05 | 2.81E-05 |
|  | CCL2 | 0.00078 | 0.000569 | 0.001223 | 3.32E-05 | 0.000411 | 3.37E-05 |
|  | IL1R1 | 1.73E-05 | 1.55E-05 | 1.74E-05 | 0.000008 | 6.06E-06 | 3.48E-06 |
|  | IL15 | 3.45E-05 | 3.97E-05 | 4.49E-05 | 1.42E-05 | 1.08E-05 | 1.85E-05 |
|  | KLF6 | 0.017 | 0.022162 | 0.020988 | 0.007105 | 0.007809 | 0.007416 |
|  | BST2 | 0.001691 | 0.001887 | 0.00168 | 0.000657 | 0.000688 | 0.000666 |
|  | IRF1 | 0.01132 | 0.011624 | 0.013071 | 0.005516 | 0.004744 | 0.005013 |
|  | ATF3 | 0.00277 | 0.002932 | 0.002994 | 0.001218 | 0.001278 | 0.00132 |
|  | TAPBP | 0.004915 | 0.004867 | 0.005171 | 0.002548 | 0.002158 | 0.002603 |
|  | B2M | 0.090718 | 0.087019 | 0.089984 | 0.045002 | 0.048709 | 0.045529 |
|  | DDR1 | 0.037767 | 0.041298 | 0.043931 | 0.023292 | 0.020018 | 0.021302 |
|  | PLAU | 0.021244 | 0.022018 | 0.023342 | 0.012017 | 0.012035 | 0.011836 |
|  | SERPINE1 | 0.011394 | 0.0119 | 0.011996 | 0.006619 | 0.006811 | 0.006528 |
|  | ISG15 | 0.005932 | 0.004932 | 0.005416 | 0.003128 | 0.003283 | 0.002923 |
|  | GM2A | 0.001171 | 0.001297 | 0.001211 | 0.000684 | 0.000735 | 0.000708 |
|  | IFNGR1 | 0.00327 | 0.003545 | 0.003393 | 0.00192 | 0.002173 | 0.001893 |
|  | RCAN1 | 0.00033 | 0.000295 | 0.000266 | 0.000174 | 0.000164 | 0.000184 |
|  | TAP1 | 0.010111 | 0.010161 | 0.011018 | 0.006456 | 0.006228 | 0.006137 |
|  | PNRC1 | 0.015778 | 0.016784 | 0.01678 | 0.010344 | 0.010417 | 0.008946 |
|  | STAT3 | 0.004042 | 0.004104 | 0.003985 | 0.002472 | 0.002549 | 0.002462 |
|  | IFIT3 | 0.005241 | 0.004754 | 0.004729 | 0.003044 | 0.003637 | 0.002663 |
|  | IFIT2 | 0.002341 | 0.002163 | 0.002043 | 0.001296 | 0.001549 | 0.001317 |
|  | FOSL2 | 0.008666 | 0.011295 | 0.010825 | 0.006799 | 0.006388 | 0.006552 |
|  | CSF1 | 0.004544 | 0.004529 | 0.004817 | 0.003237 | 0.002902 | 0.00288 |
|  | IFNAR1 | 0.00238 | 0.002251 | 0.002207 | 0.001462 | 0.001634 | 0.001564 |
|  | KDM6B | 0.003519 | 0.003971 | 0.004251 | 0.003107 | 0.002591 | 0.002411 |
|  | PSEN1 | 0.001281 | 0.001327 | 0.001259 | 0.000953 | 0.000956 | 0.000933 |
|  | CSTB | 0.035693 | 0.033557 | 0.033076 | 0.02657 | 0.024149 | 0.025048 |
|  | STAT1 | 0.00394 | 0.003702 | 0.003605 | 0.002932 | 0.002972 | 0.002983 |
|  | CLU | 0.015678 | 0.014271 | 0.015368 | 0.012178 | 0.011703 | 0.011972 |
|  | NFKB2 | 0.017959 | 0.016302 | 0.016969 | 0.014122 | 0.013154 | 0.013316 |
|  | IL15RA | 0.001253 | 0.001218 | 0.001261 | 0.000952 | 0.001078 | 0.001033 |

| **Table S4** **The clinicopathological characteristics of patients with ESCC** | | | | | | | |
| --- | --- | --- | --- | --- | --- | --- | --- |
| **Clinical and pathological indexes** | **Case No.** | 5-year OS (%) | *P** |  | 5-year DFS (%) | *P** |  |
| Specimens | 301 |  |  |  |  |  |  |
| Mean age | 58.6 |  |  |  |  |  |  |
| Age (year) |  |  |  |  |  |  |  |
| <59 | 157 | 45.5 | 0.086 |  | 43.3 | 0.115 |  |
| ≥59 | 144 | 38.6 |  |  | 35.9 |  |  |
| Gender |  |  |  |  |  |  |  |
| Male | 229 | 41.2 | 0.385 |  | 38.8 | 0.164 |  |
| Female | 72 | 45.5 |  |  | 42.7 |  |  |
| Tumor location |  |  |  |  |  |  |  |
| upper | 24 | 43.3 | 0.886 |  | 39.7 | 0.957 |  |
| middle | 155 | 42.2 |  |  | 40.2 |  |  |
| lower | 122 | 42.0 |  |  | 39.2 |  |  |
| Histologic grade |  |  |  |  |  |  |  |
| G1 | 73 | 50.7 | 0.110 |  | 45.8 | 0.204 |  |
| G2 | 199 | 41.4 |  |  | 39.2 |  |  |
| G3 | 29 | 27.6 |  |  | 27.6 |  |  |
| Primary tumor |  |  |  |  |  |  |  |
| T1 | 5 | 60.0 | 0.776 |  | 60.0 | 0.767 |  |
| T2 | 26 | 45.0 |  |  | 45.1 |  |  |
| T3 | 268 | 41.6 |  |  | 39.1 |  |  |
| T4 | 2 | 50.0 |  |  | 0.0 |  |  |
| Regional lymph node | | | | | | |  |
| N0 | 146 | 58.0 | 0.000 |  | 55.6 | 0.007 |  |
| N1 | 102 | 36.5 |  |  | 33.3 |  |  |
| N2 | 42 | 13.4 |  |  | 11.9 |  |  |
| N3 | 11 | 0.0 |  |  | 0.0 |  |  |
| pTNM-stage |  |  |  |  |  |  |  |
| I(IA+IB) | 16 | 55.6 | 0.000 |  | 56.3 | 0.000 |  |
| II(IIA+IIB) | 139 | 56.6 |  |  | 54.8 |  |  |
| III(IIIA+IIIB+IIIC) | 146 | 27.0 |  |  | 23.4 |  |  |
| *Log-rank test of Kaplan Meier method; *P* <0.05 was considered significant. | | | | | | | |
| All patients underwent surgical treatment. OS: overall survival; DFS: disease free survival | | | | | | | |

**Table S5** **Primer sequences for qRT-PCR**

| Name | Forward primer (5’-3’) | Reverse primer (5’-3’) |
| --- | --- | --- |
| SOCS3 | GTAGGACGGAGACTTCGATTC | CTTGCTGTGGGTGACCATG |
| MYC | AGCTGCTTAGACGCTGGATT | CTCCTCGTCGCAGTAGAAAT |
| CCNA2 | TGCATTGGTCCCTCTTGAT | TAACCTCCATTTCCCTAAG |
| CCND1 | CATCTACACCGACAACTCCA | CACAGAGGGCAACGAAGGT |
| CCNB1 | GTTGGTTTCTGCTGGGTGTA | CATGTTGATCTTCGCCTTAT |
| CCND2 | CTGGAGTGGGAACTGGTGGT | ATCATCGACGGTGGGTACAT |
| CDC45 | TTGAAGTTCCCGCCTATGAA | CATGGTTTGCTCCACTATCTC |
| CCL2 | AGAATCACCAGCAGCAAG | GGAATCCTGAACCCACTT |
| IL1R1 | CTCCAGGATTCATCAACACA | GTAGGCTCATTCTCCACAA |
| IFIT3 | GCAGGGAAACAGCCATCATG | CAAGCCCAGGAGGCAAAAGT |
| TNFRSF9 | TGCTCTTCCTGCTGTTCTTC | CACAGTTCACATCCTCCTTC |
| ACTB | TCCCTGGAGAAGAGCTACGA | CTGTGTTGGCGTACAGGTCT |
| SOCS3-primer1 | CTCCGCCTGCCAAAGGGAAG | TGTGCTGGGGACTCAGTGAGA |
| SOCS3-primer2 | AGAGAGGCAGGGGGTCACAT | CAGGCTGGGGTCAGACCTG |

**Table S6 The plasmids and oligonucleotides used in this study**

| Plasmid | Source |
| --- | --- |
| pLVX-MCS-TagBFP-Puro | Miaolingbio (Wuhan, China), #P18446 |
| pLVX-DsRed-Monomer-N1 | Miaolingbio (Wuhan, China), # P0252 |
| pLVX N-Flag ELK1 | This paper |
| pLVX N-Flag SOCS3 | This paper |
| pBOBi ERK2 C-HA | [2] |
| pGL3 SOCS3-promoter | This paper |
| pGL3 SOCS3-promoter-mut-a | This paper |
| pGL3 SOCS3-promoter-mut-b | This paper |
| pGL3 SOCS3-promoter-mut-a+b | This paper |
| pcDNA3.3 gRNA-ERK1 | [2] |
| pcDNA3.3 gRNA-ERK2 | [2] |
| pcDNA3.3 gRNA-STAT3 | This paper |
| pLKO.1 | Addgene plasmid: #10878 |

| Oligonucleotides | Forward primer (5’-3’) | Reverse primer (5’-3’) |
| --- | --- | --- |
| ERK2 | ATGGCGGCGGCGGCGGCGGCGGG | TTAAGATCTGTATCCTGGCTGGAATCT |
| ELK1 | ATGGACCCATCTGTGACGCT | TCTTGGCTTCTGGGCCCTGGGA |
| SOCS3 | ATGGTCACCCACAGCAAGTTTC | TTAAAGCGGGGCATCGTACTG |
| gRNA: ERK1 | CACCGCGACCCCCTCGGTTCTACG | AAACCGTAGAACCGAGGGGGTCGC |
| gRNA: ERK2 | CACCGGCGGGCAGGTGTTCGACG | AAACACGTCGAACACCTGCCCGCC |
| gRNA: STAT3 | CACCGCAGCTTGACACACGGTACC | AAACGGTACCGTGTGTCAAGCTGC |
| shSOCS3-1:  shSOCS3-2: | CCGGCCGCTTCGACTGCGTGCTCAACTCGAGTTGAGCACGCAGTCGAAGCGGTTTTTG  CCGGCGGCTTCTACTGGAGCGCAGTCTCGAGACTGCGCTCCAGTAGAAGCCGTTTTTG | AATTCAAAAACCGCTTCGACTGCGTGCTCAACTCGAGTTGAGCACGCAGTCGAAGCGG  AATTCAAAAACGGCTTCTACTGGAGCGCAGTCTCGAGACTGCGCTCCAGTAGAAGCCG |
| SOCS3 promoter | TTGACCTCCCTGACTCAAGCGA | CAGGCTGGGGTCAGACCTG |

**Reference**

1 Liu W, Xie L, He YH, Wu ZY, Liu LX, Bai XF *et al*. Large-scale and high-resolution mass spectrometry-based proteomics profiling defines molecular subtypes of esophageal cancer for therapeutic targeting. *Nat Commun* 2021; 12: 4961.

2 Zheng ZY, Yang PL, Li RY, Liu LX, Xu XE, Liao LD *et al*. STAT3beta disrupted mitochondrial electron transport chain enhances chemosensitivity by inducing pyroptosis in esophageal squamous cell carcinoma. *Cancer Lett* 2021.

3 Zhan XH, Jiao JW, Zhang HF, Xu XE, He JZ, Li RL *et al*. LOXL2 Upregulates Phosphorylation of Ezrin to Promote Cytoskeletal Reorganization and Tumor Cell Invasion. *Cancer Res* 2019; 79: 4951-4964.

4 Zou HY, Lv GQ, Dai LH, Zhan XH, Jiao JW, Liao LD *et al*. A truncated splice variant of human lysyl oxidase-like 2 promotes migration and invasion in esophageal squamous cell carcinoma. *Int J Biochem Cell Biol* 2016; 75: 85-98.

5 Hernandez-Segura A, Brandenburg S, Demaria M. Induction and Validation of Cellular Senescence in Primary Human Cells. *J Vis Exp* 2018.
