# Supplementary material for: Blocking STAT3 signaling augments MEK/ERK inhibitor efficacy in esophageal squamous cell carcinoma
Source: Cell Death Dis. 2022 May 25;13(5):496. doi: 10.1038/s41419-022-04941-3 (PMC9132929; doi:10.1038/s41419-022-04941-3)

Figure 1D

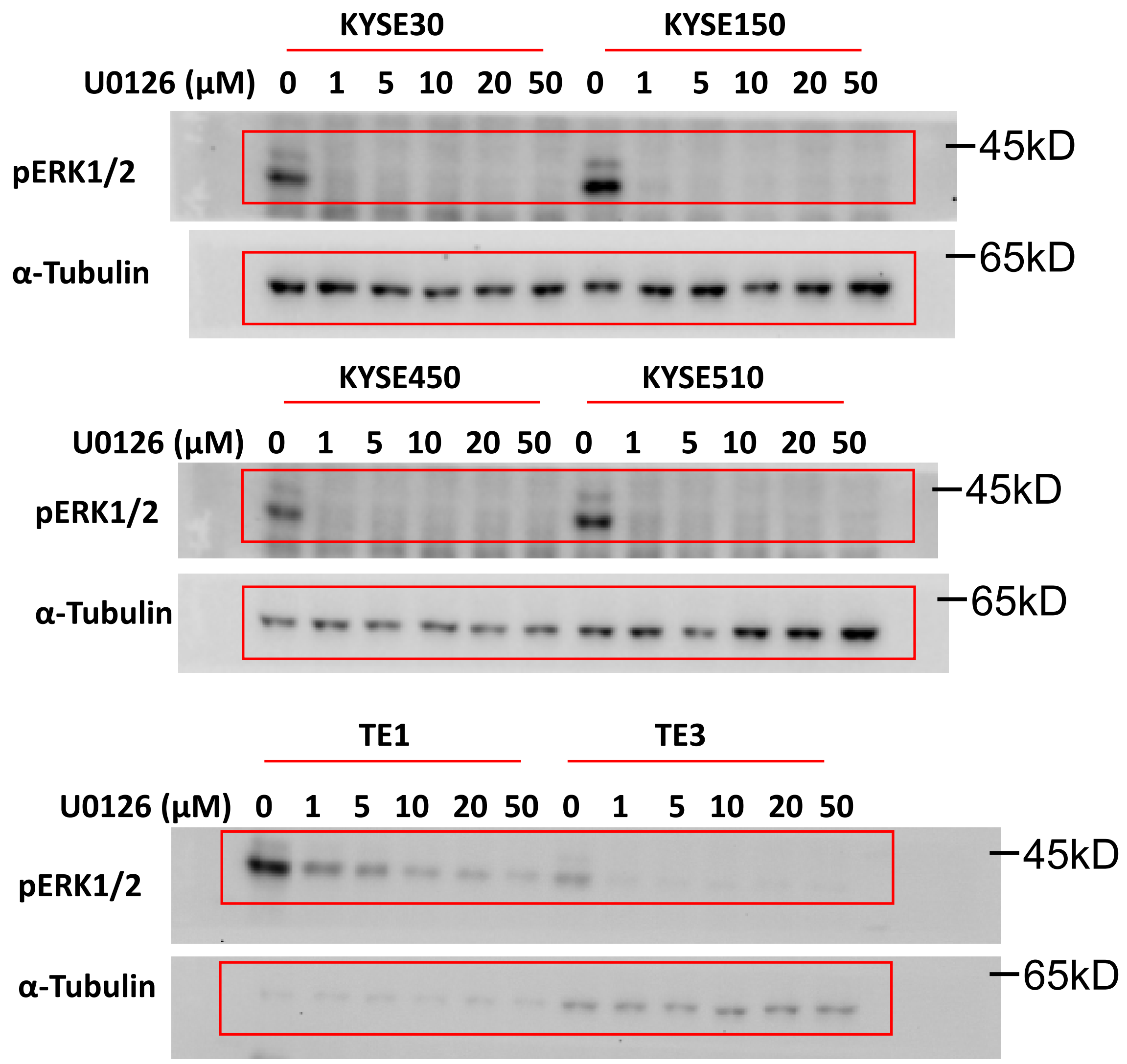

Figure 2C

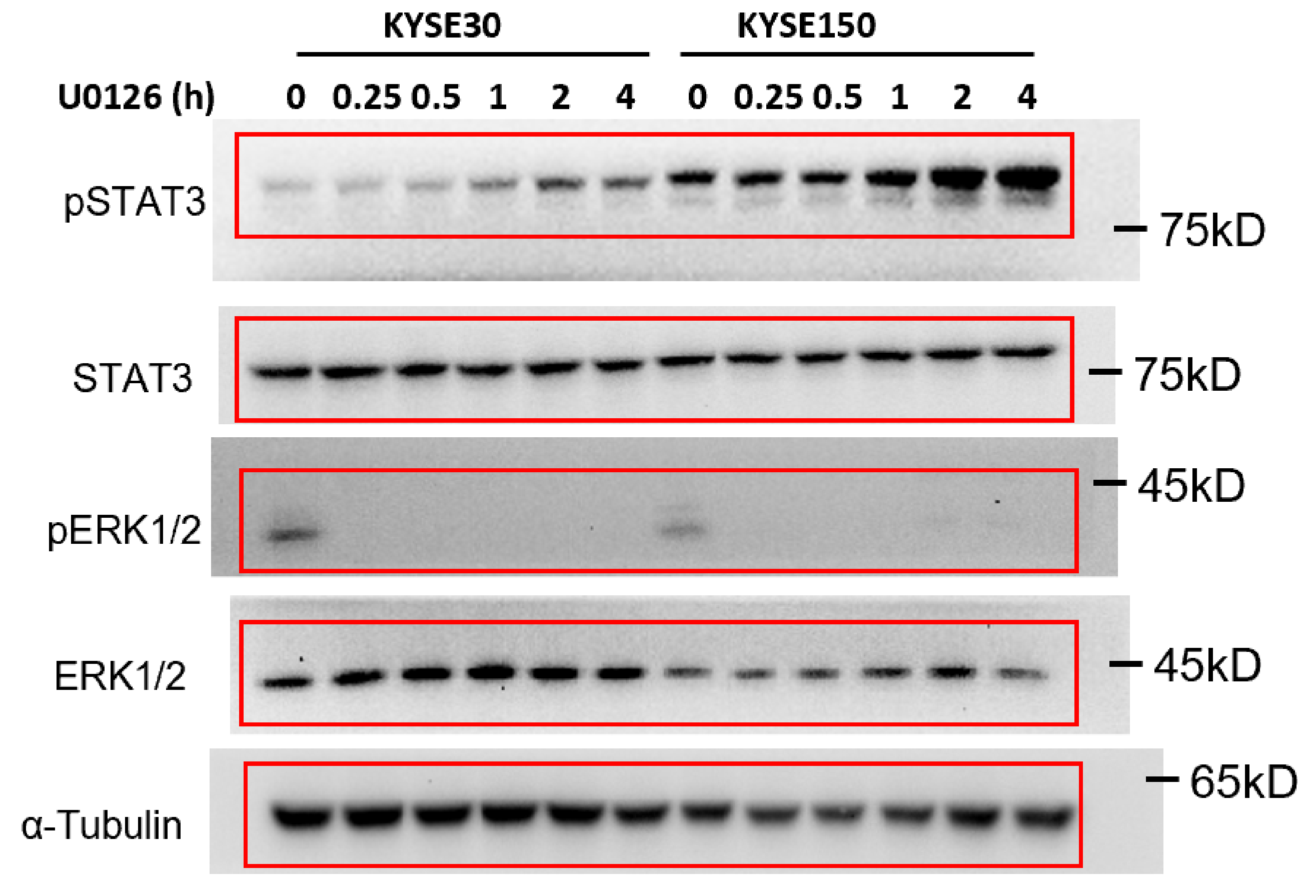

Figure 2E

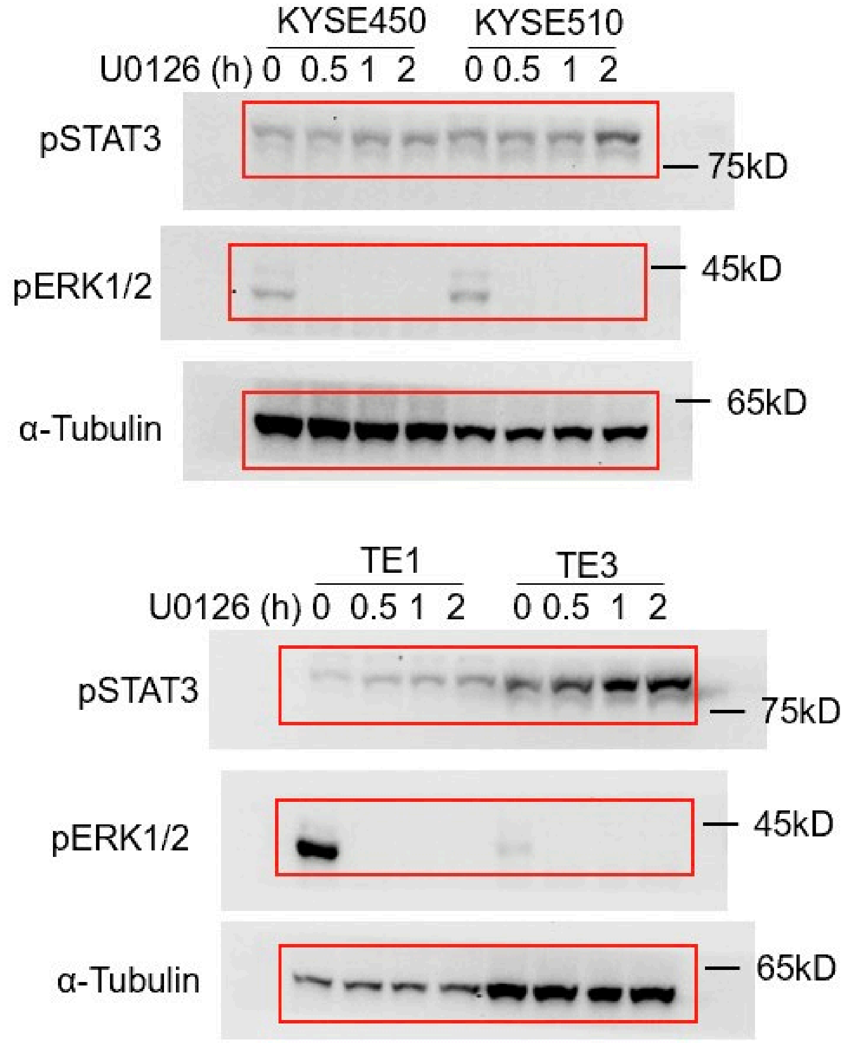

Figure 2F left

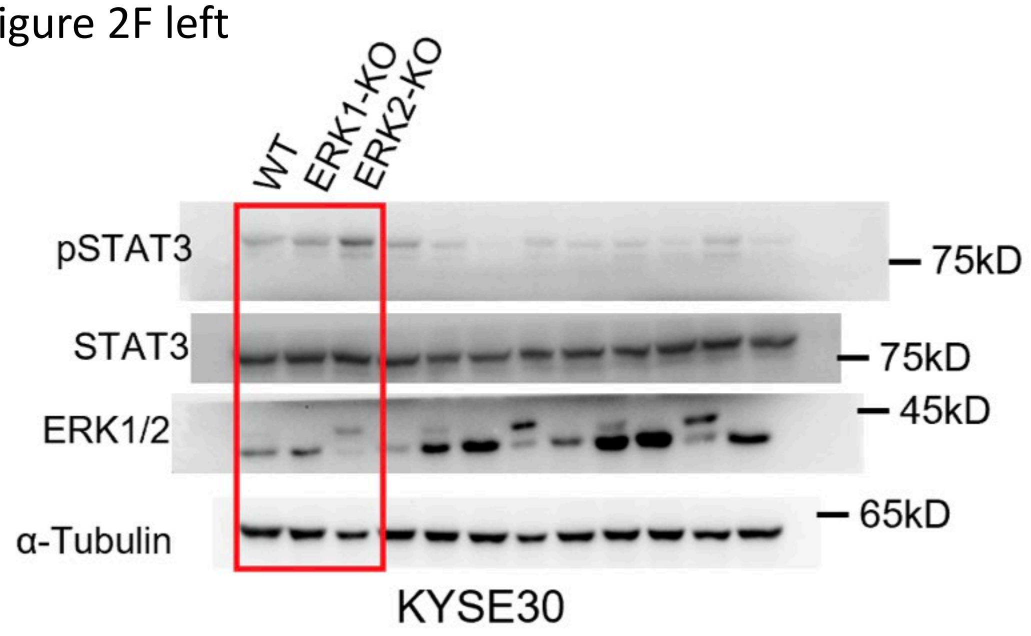

Figure 2F right

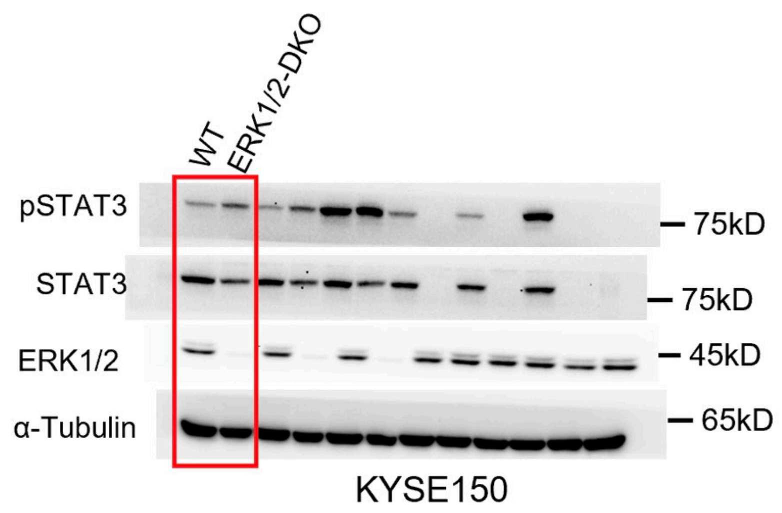

Figure 2G left

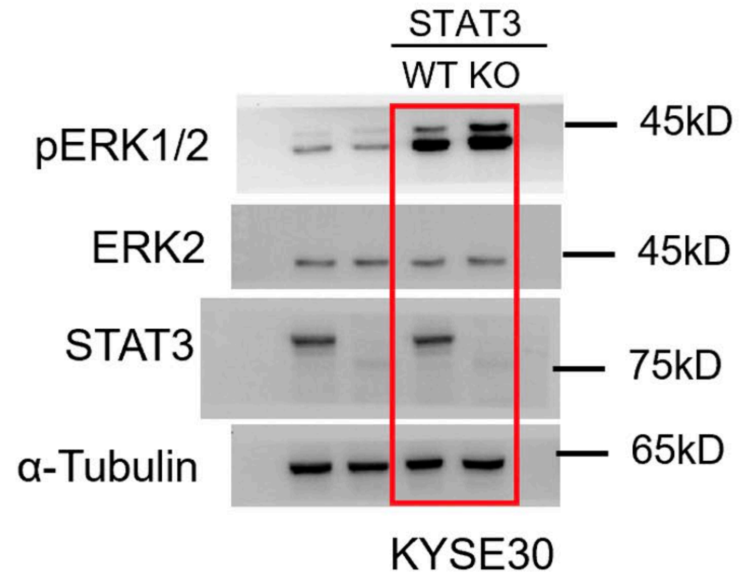

Figure 2G right

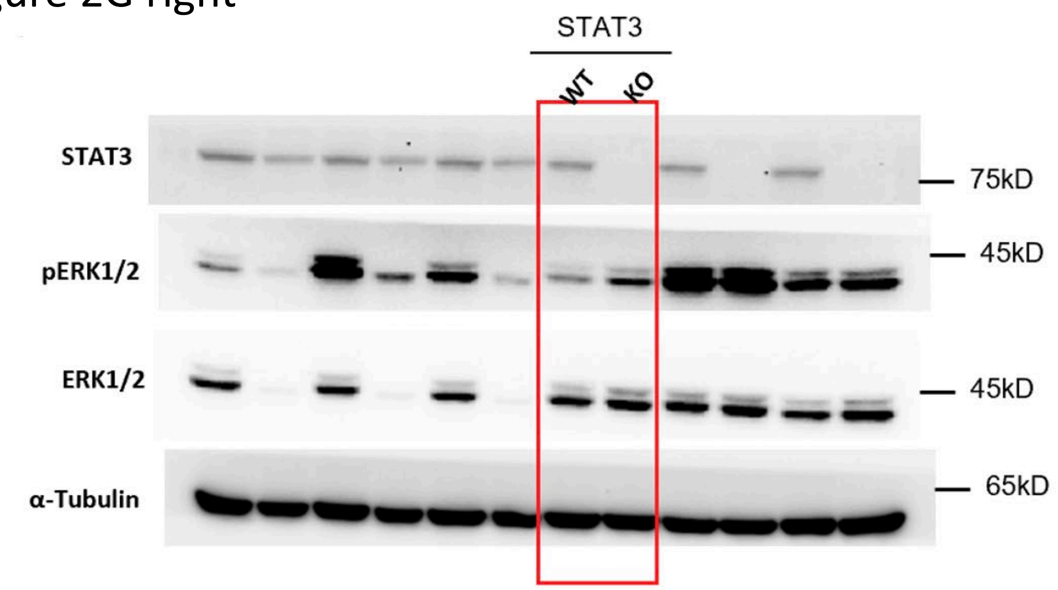

Figure 2H

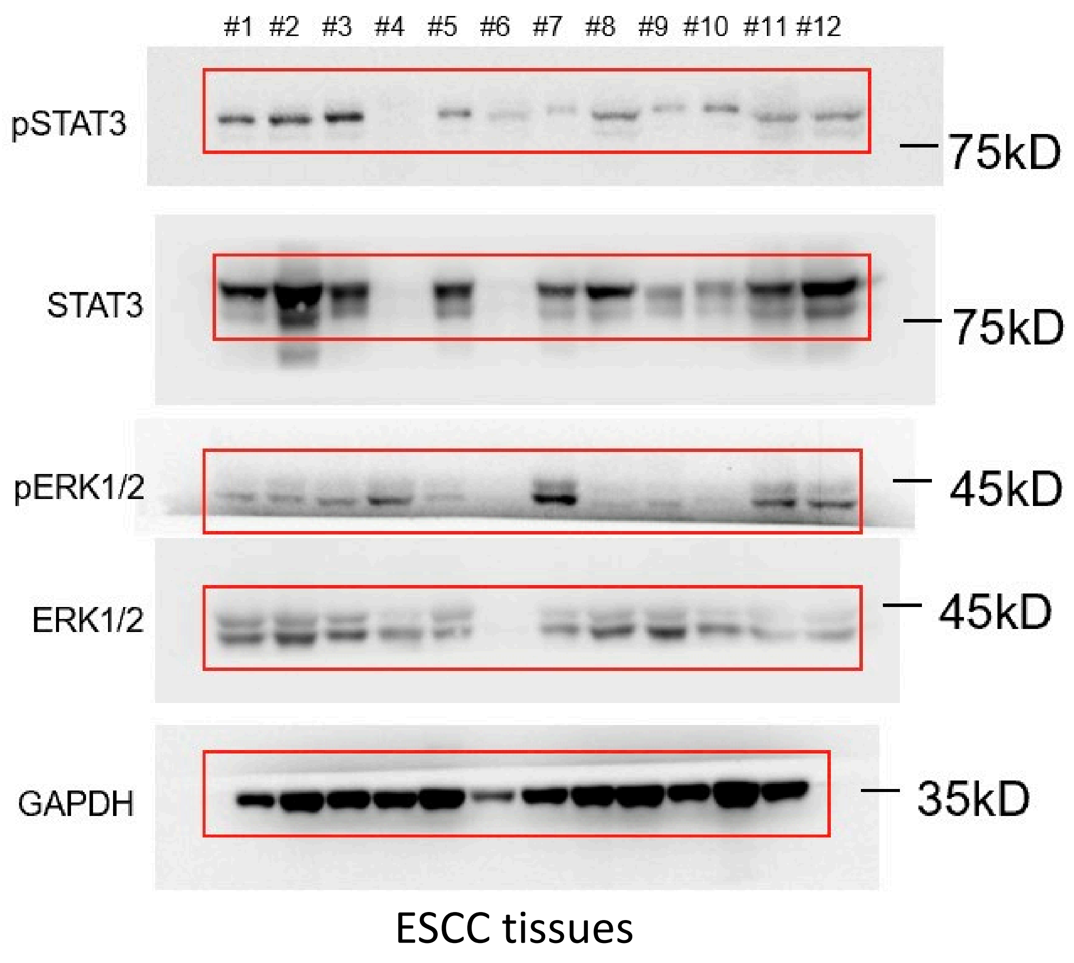

Figure 3A

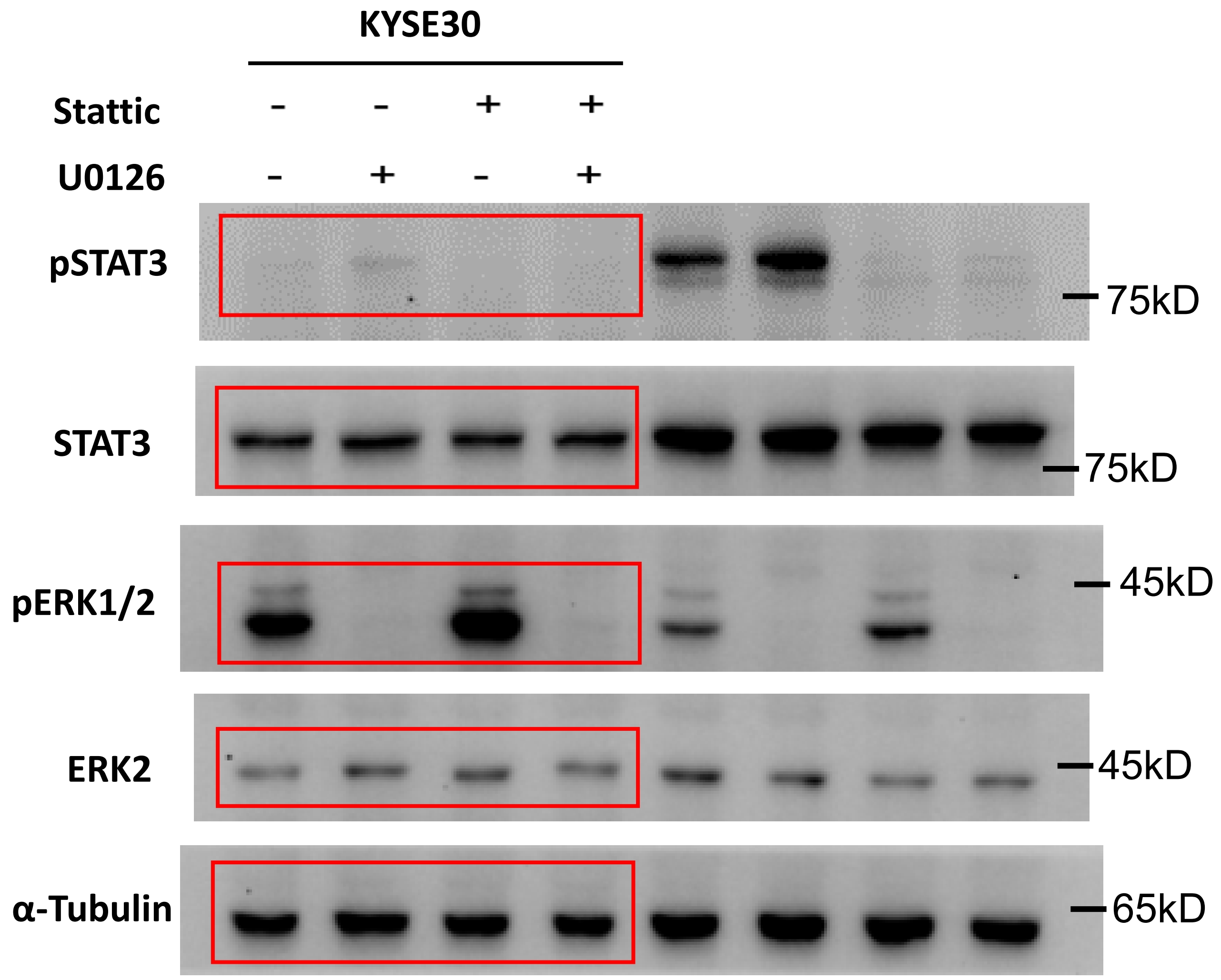

Figure 3B

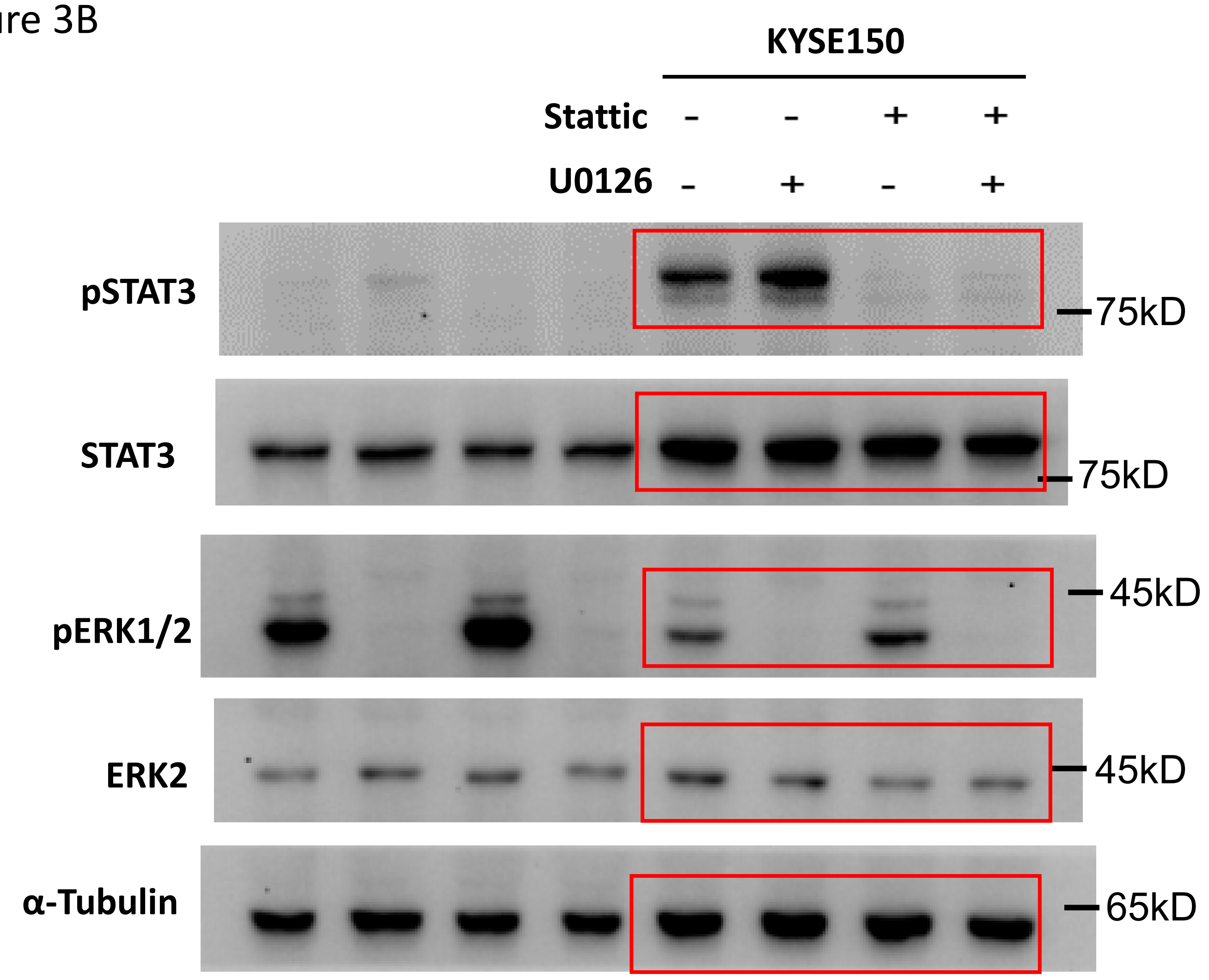

Figure 4A left

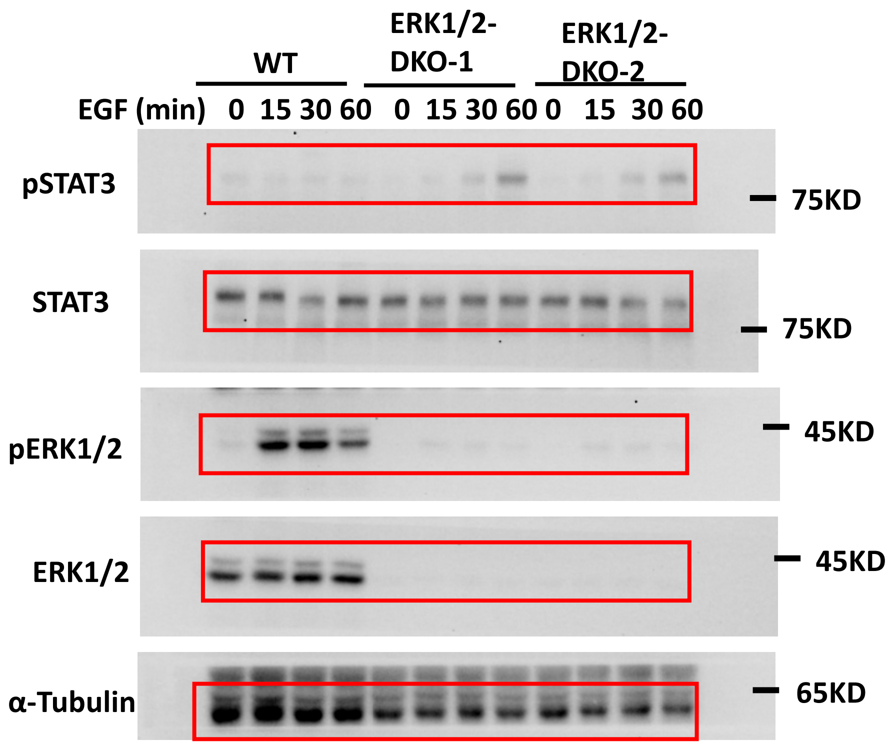

Figure 4A middle

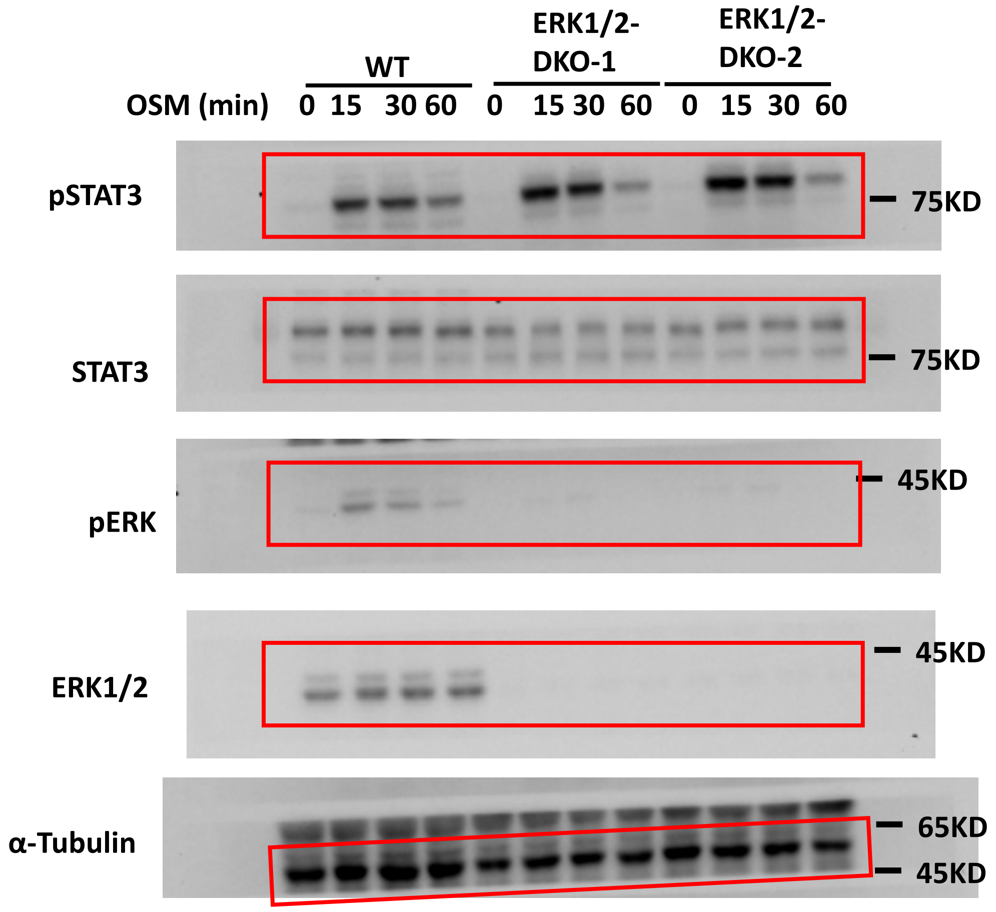

Figure 4A right

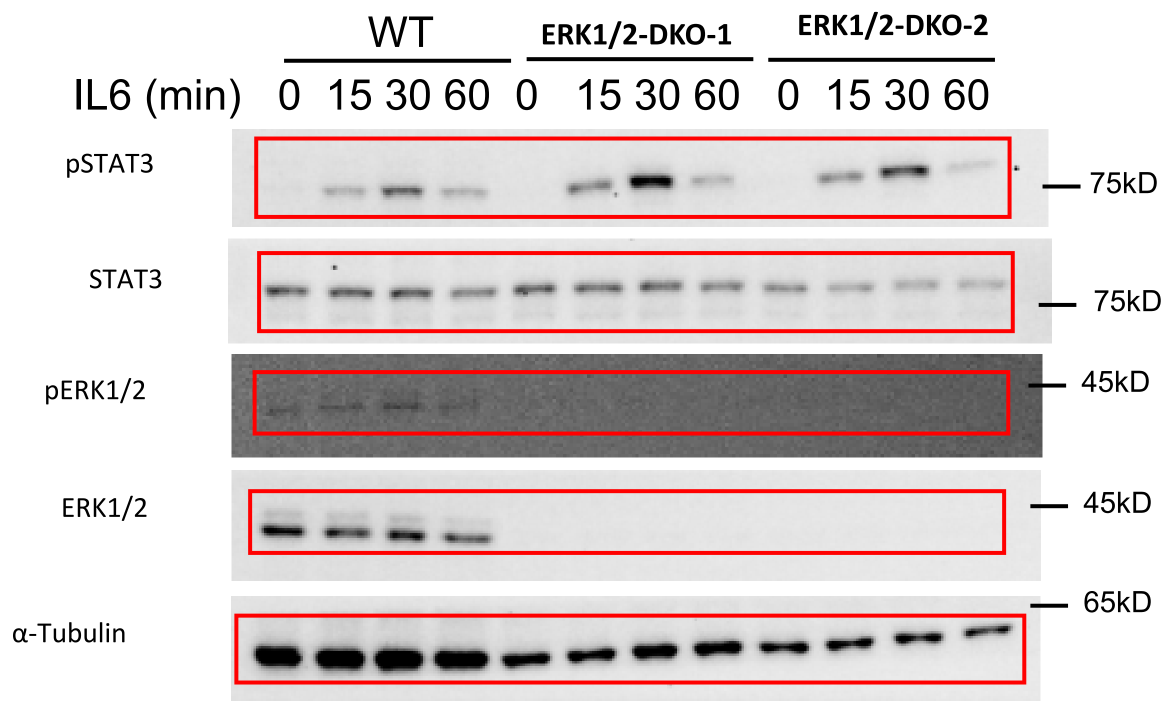

Figure 4B

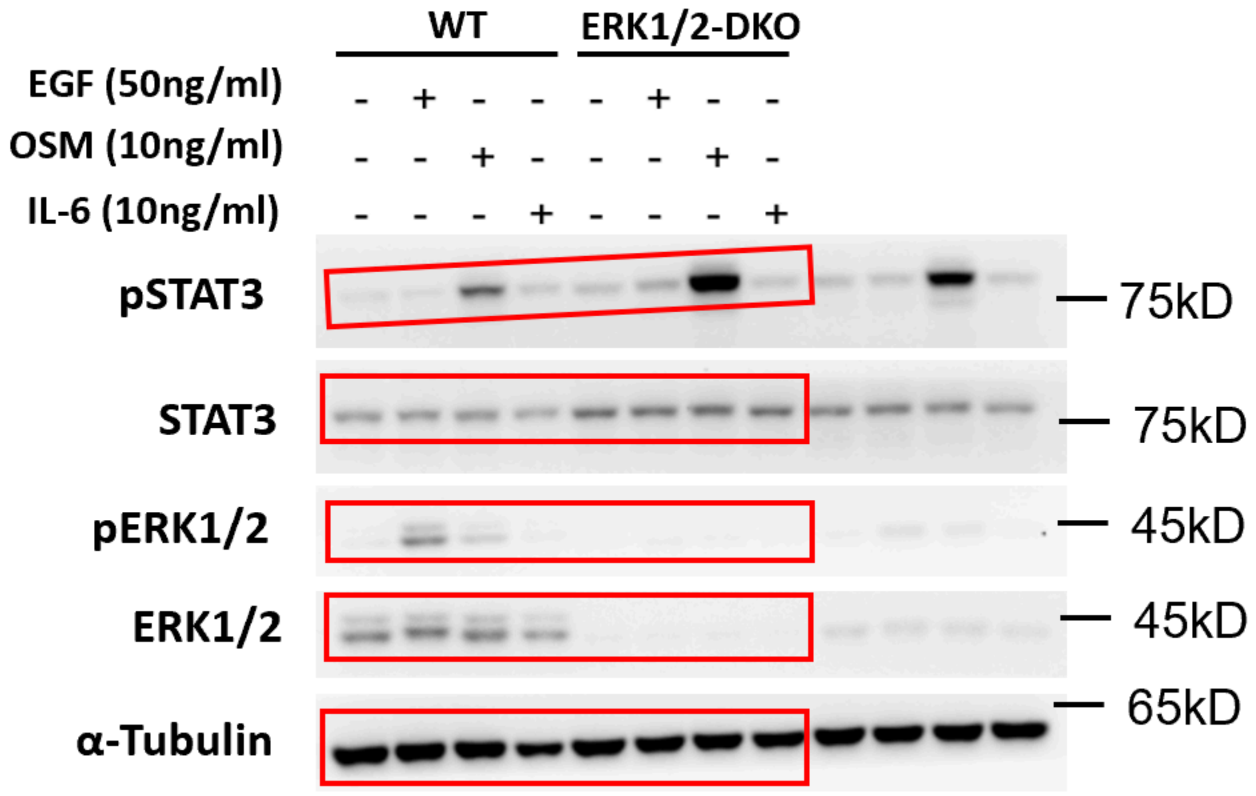

Figure 4E

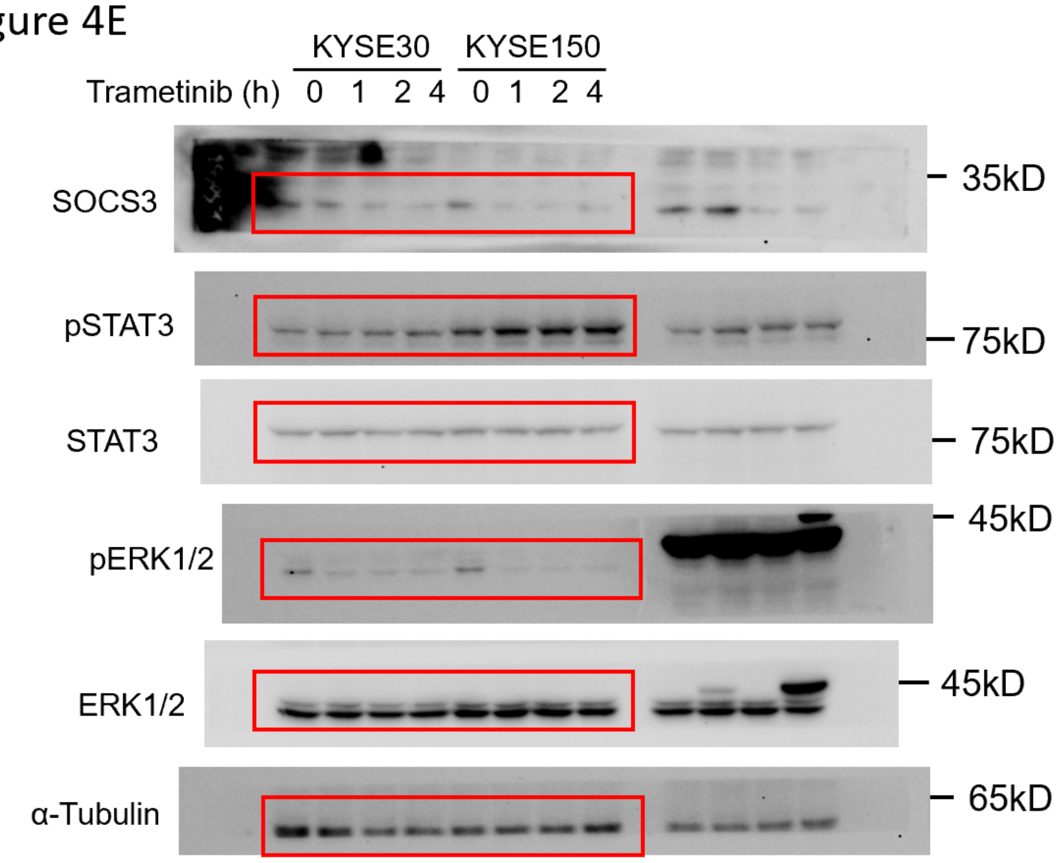

Figure 4F

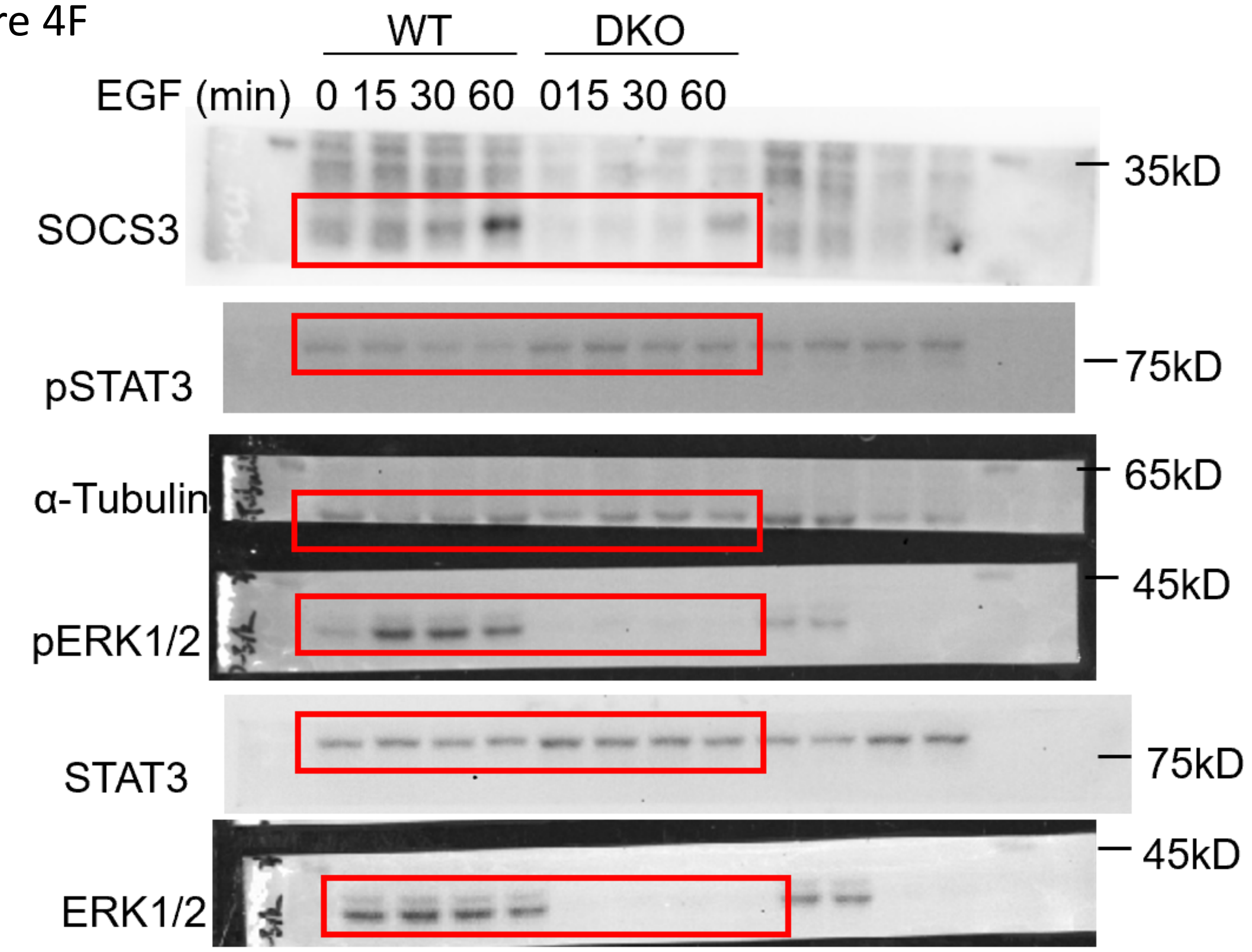

Figure 4G

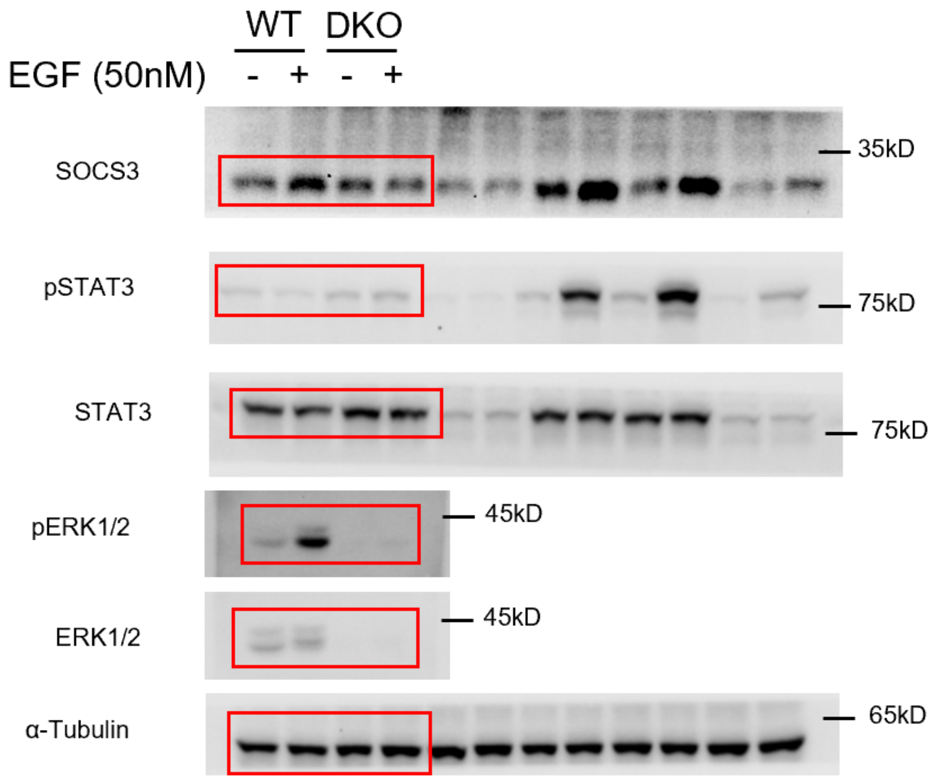

Figure 4H

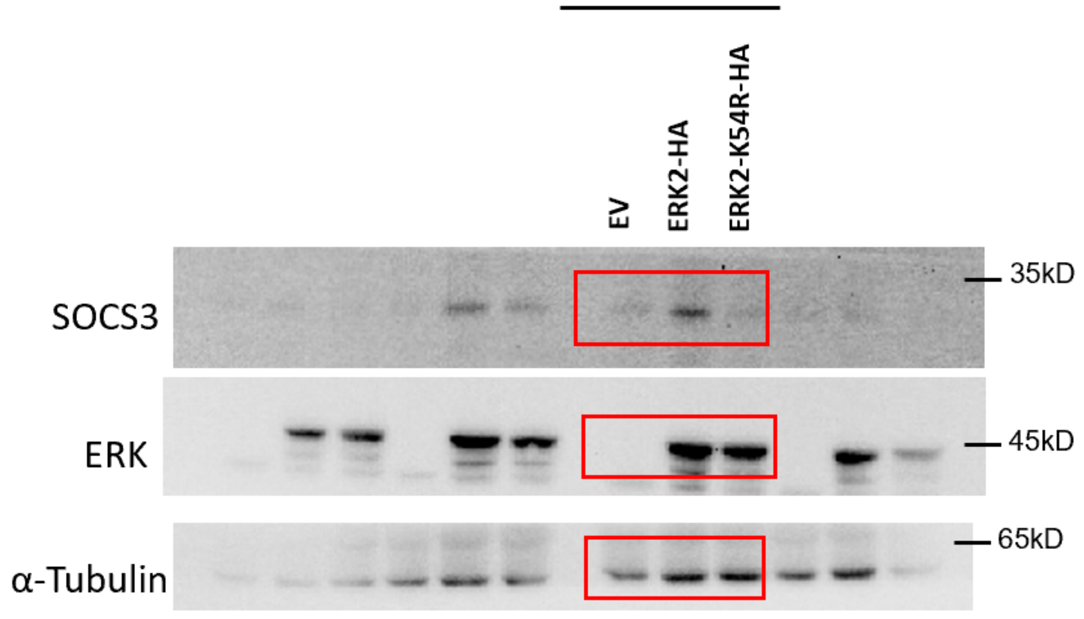

Figure 4J

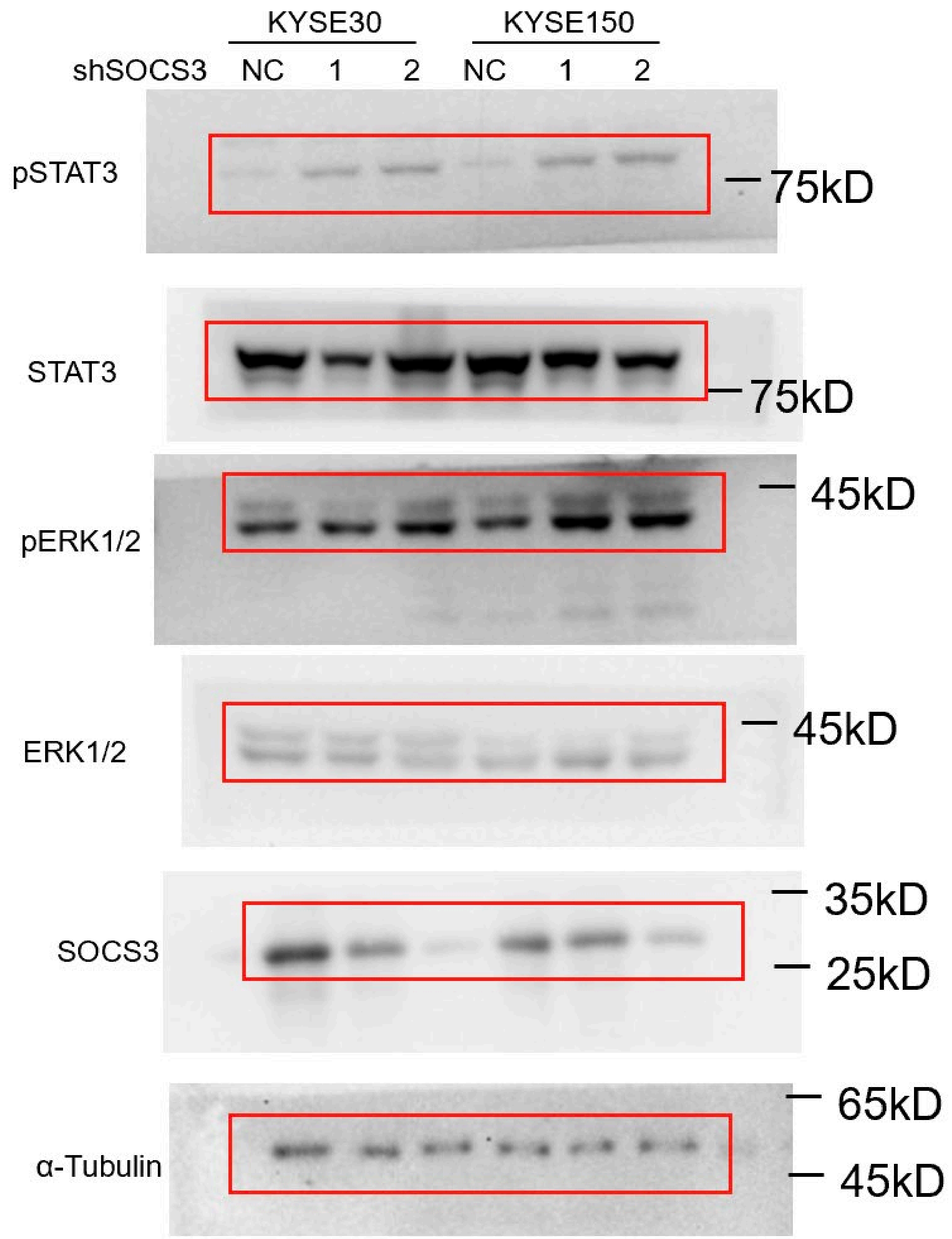

Figure 4I left

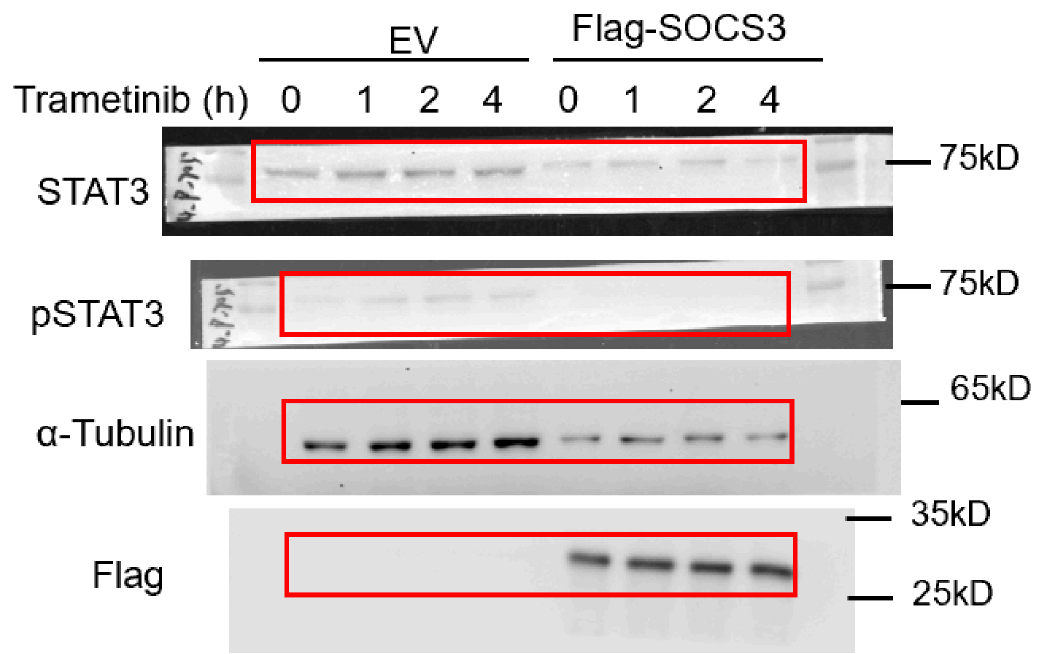

Figure 4I right

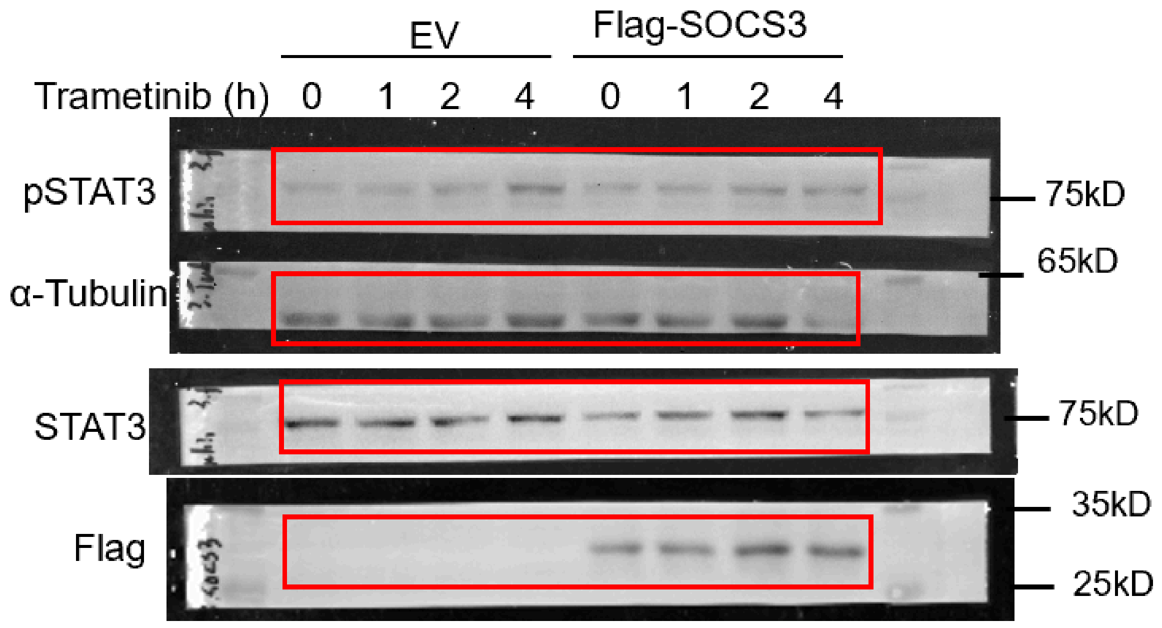

Figure 5H

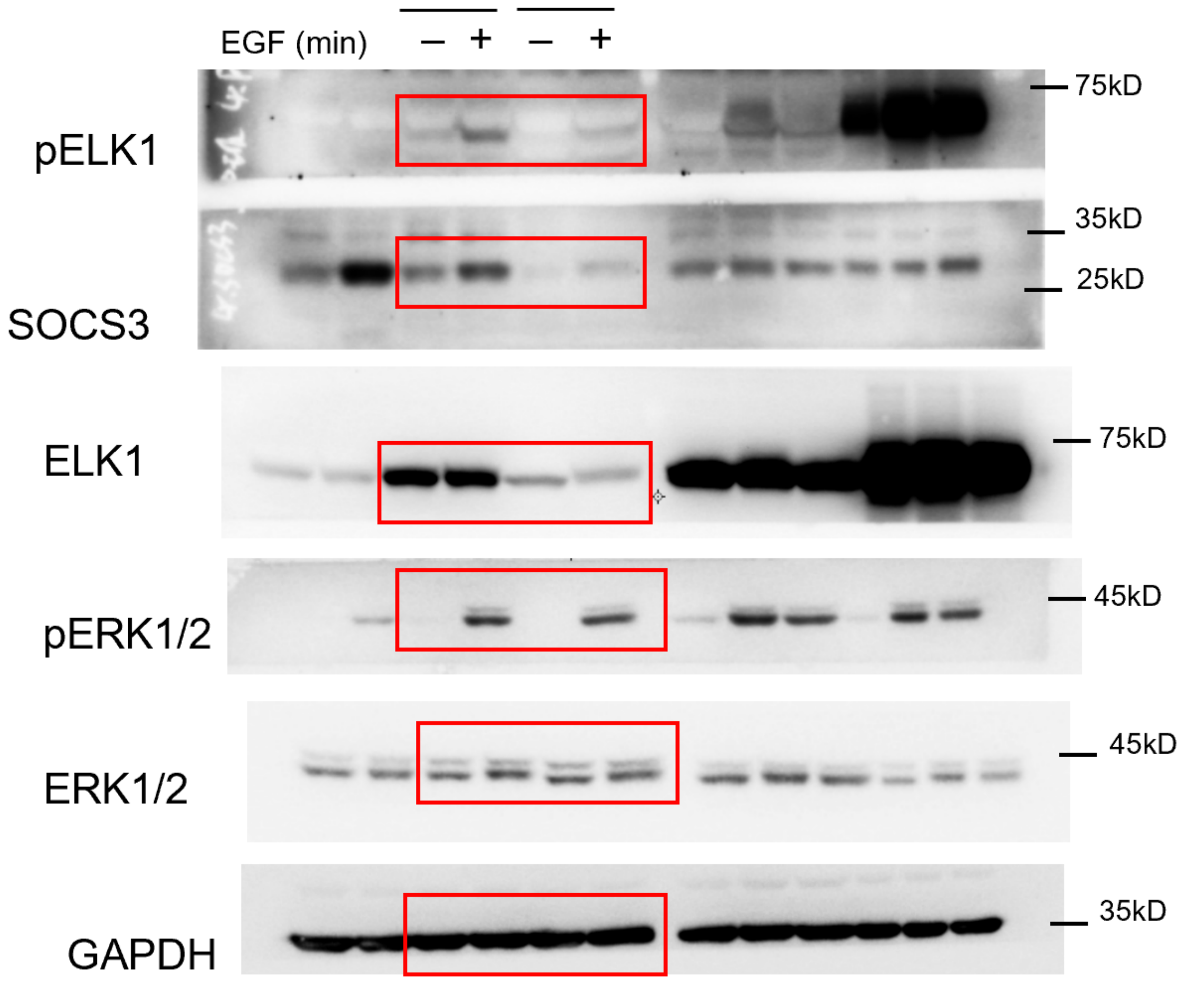

Figure 5I

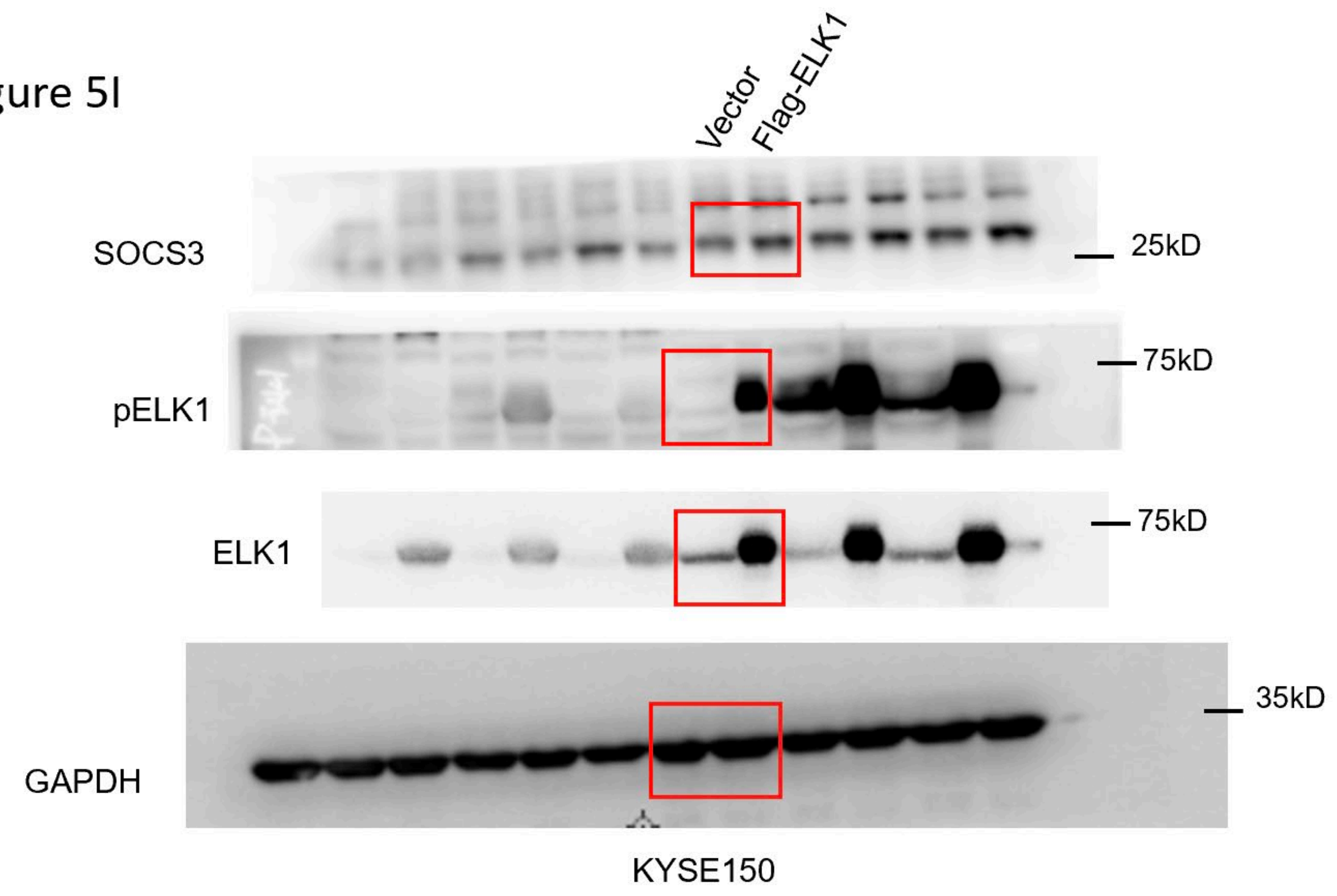

Figure 5J

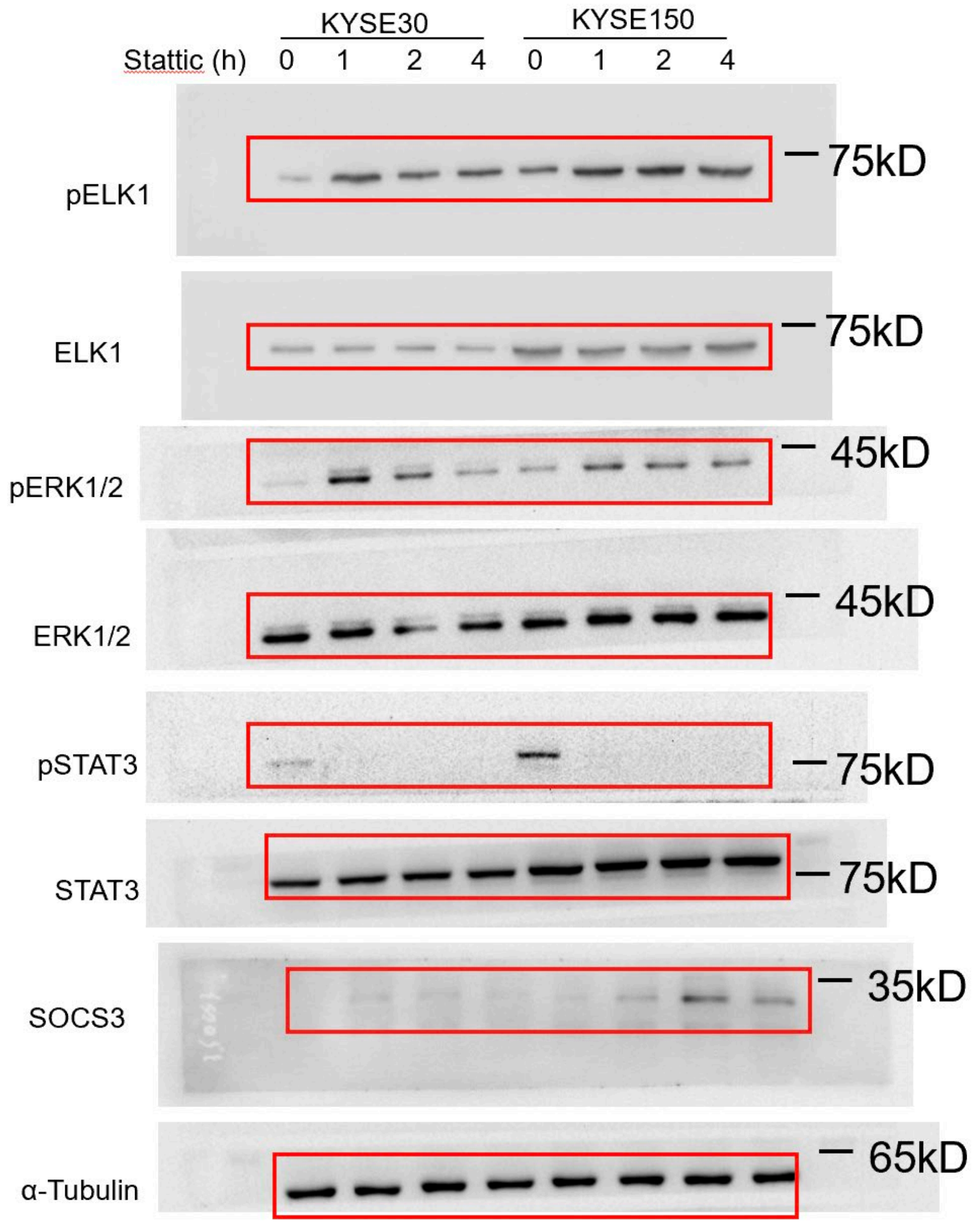

Figure 6F

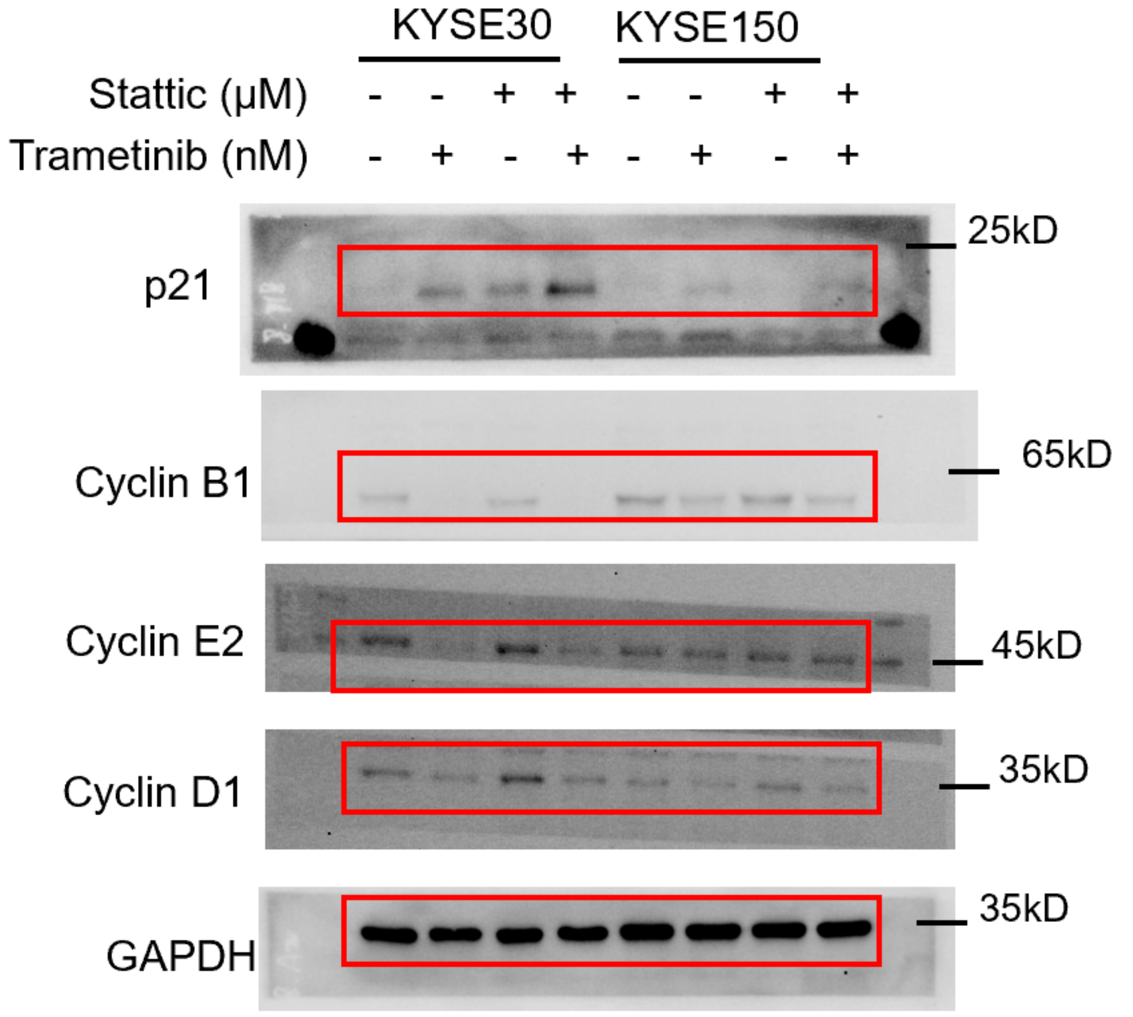

Figure 7D

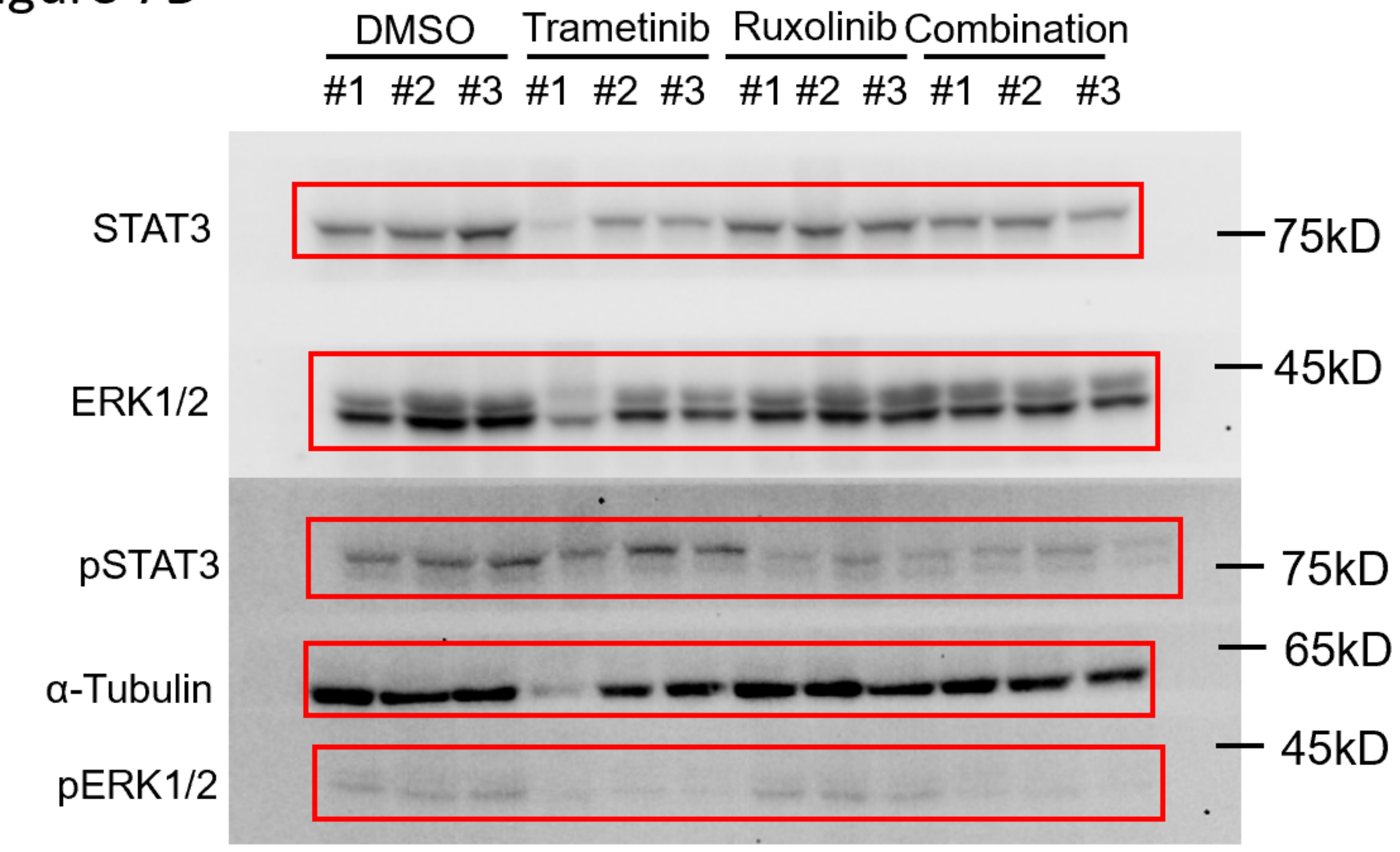

Figure S1B

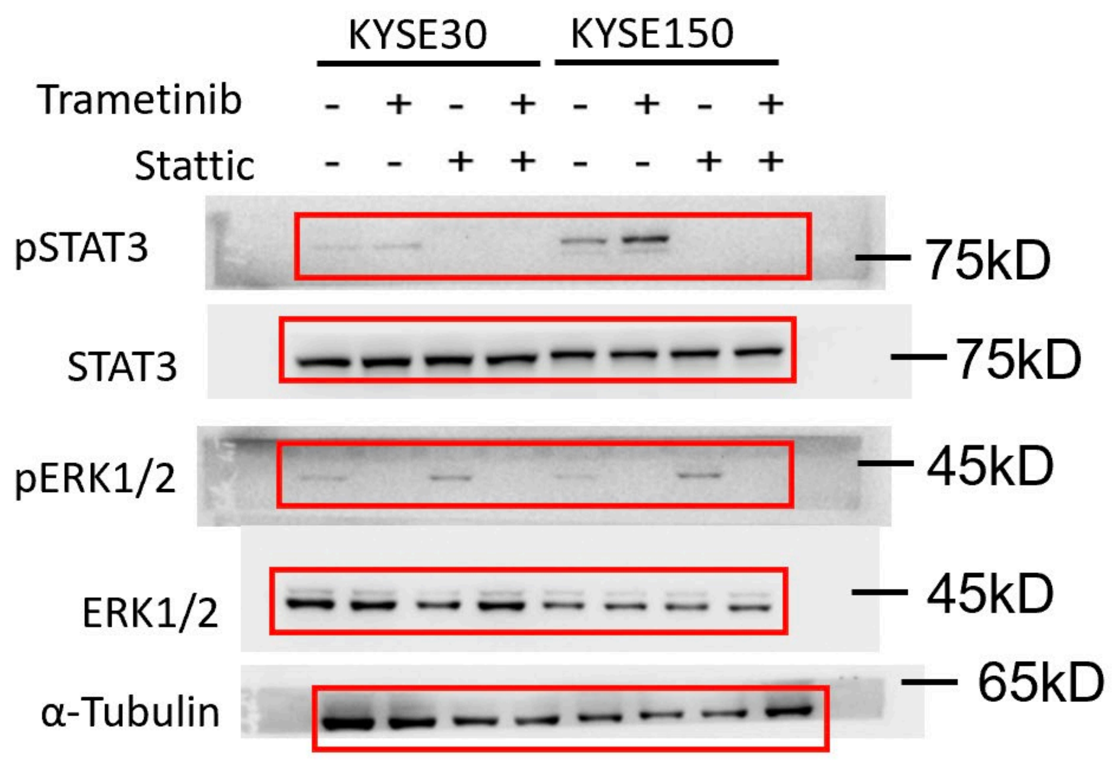

Figure S1D

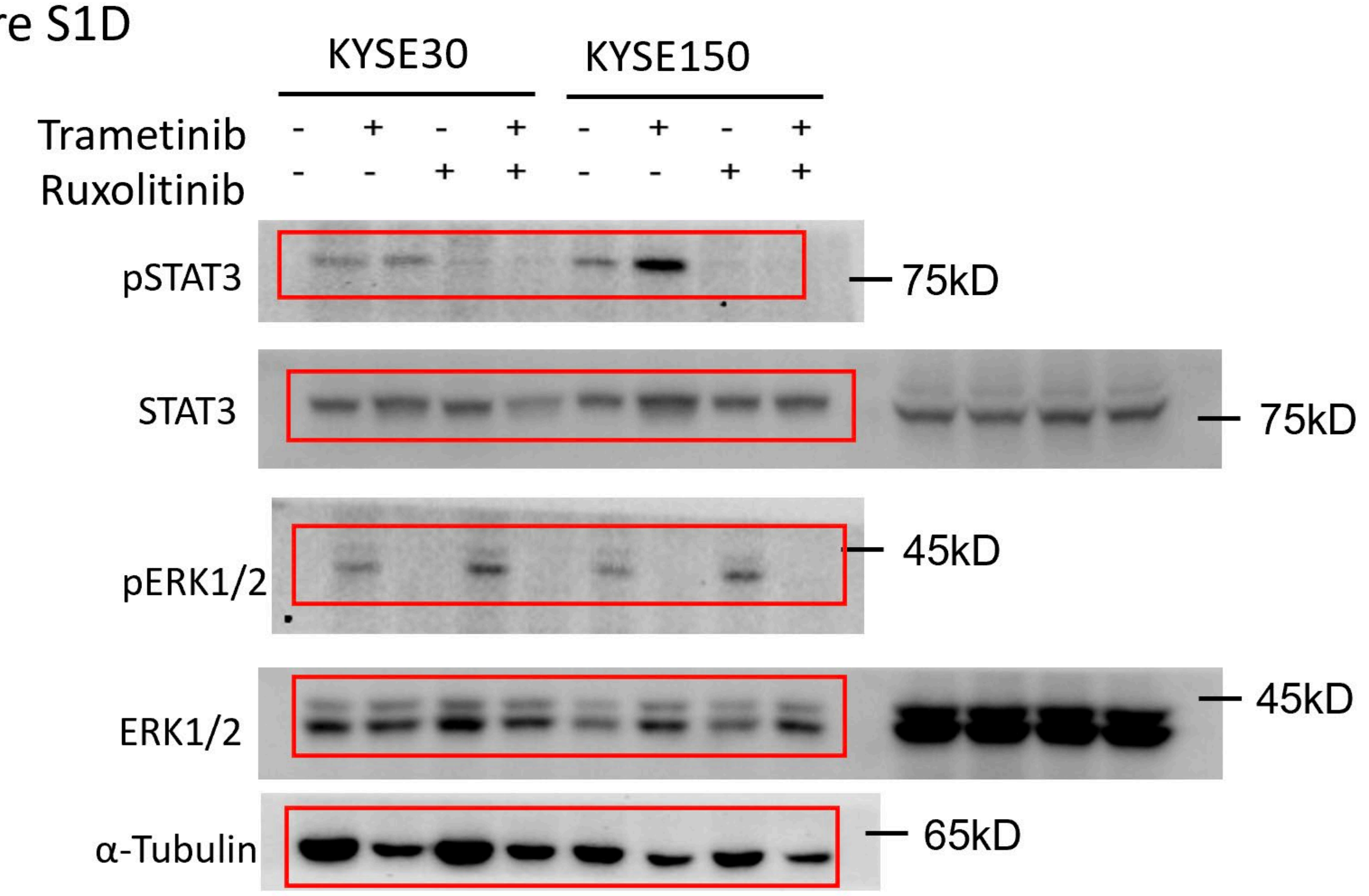

Supplement: Supplementary file 3 — Original Data File [file 41419_2022_4941_MOESM3_ESM.pdf]
